# The future of targeted kinase inhibitors in melanoma

**DOI:** 10.1016/j.pharmthera.2022.108200

**Published:** 2022-05-02

**Authors:** Signe Caksa, Usman Baqai, Andrew E. Aplin

**Affiliations:** a Department of Cancer Biology, Thomas Jefferson University, Philadelphia, PA 19107, USA; b Sidney Kimmel Cancer Center, Thomas Jefferson University, Philadelphia, PA 19107, USA

**Keywords:** Cancer, Melanoma, Targeted therapy, Kinase inhibitors, Immune checkpoint inhibitors, Combination therapy

## Abstract

Melanoma is a cancer of the pigment-producing cells of the body and its incidence is rising. Targeted inhibitors that act against kinases in the MAPK pathway are approved for *BRAF*-mutant metastatic cutaneous melanoma and increase patients’ survival. Response to these therapies is limited by drug resistance and is less durable than with immune checkpoint inhibition. Conversely, rare melanoma subtypes have few therapeutic options for advanced disease and MAPK pathway targeting agents show minimal anti-tumor effects. Nevertheless, there is a future for targeted kinase inhibitors in melanoma: in new applications such as adjuvant or neoadjuvant therapy and in novel combinations with immunotherapies or other targeted therapies. Pre-clinical studies continue to identify tumor dependencies and their corresponding actionable drug targets, paving the way for rational targeted kinase inhibitor combinations as a personalized medicine approach for melanoma.

## 1. Introduction

**Fig. 1.**
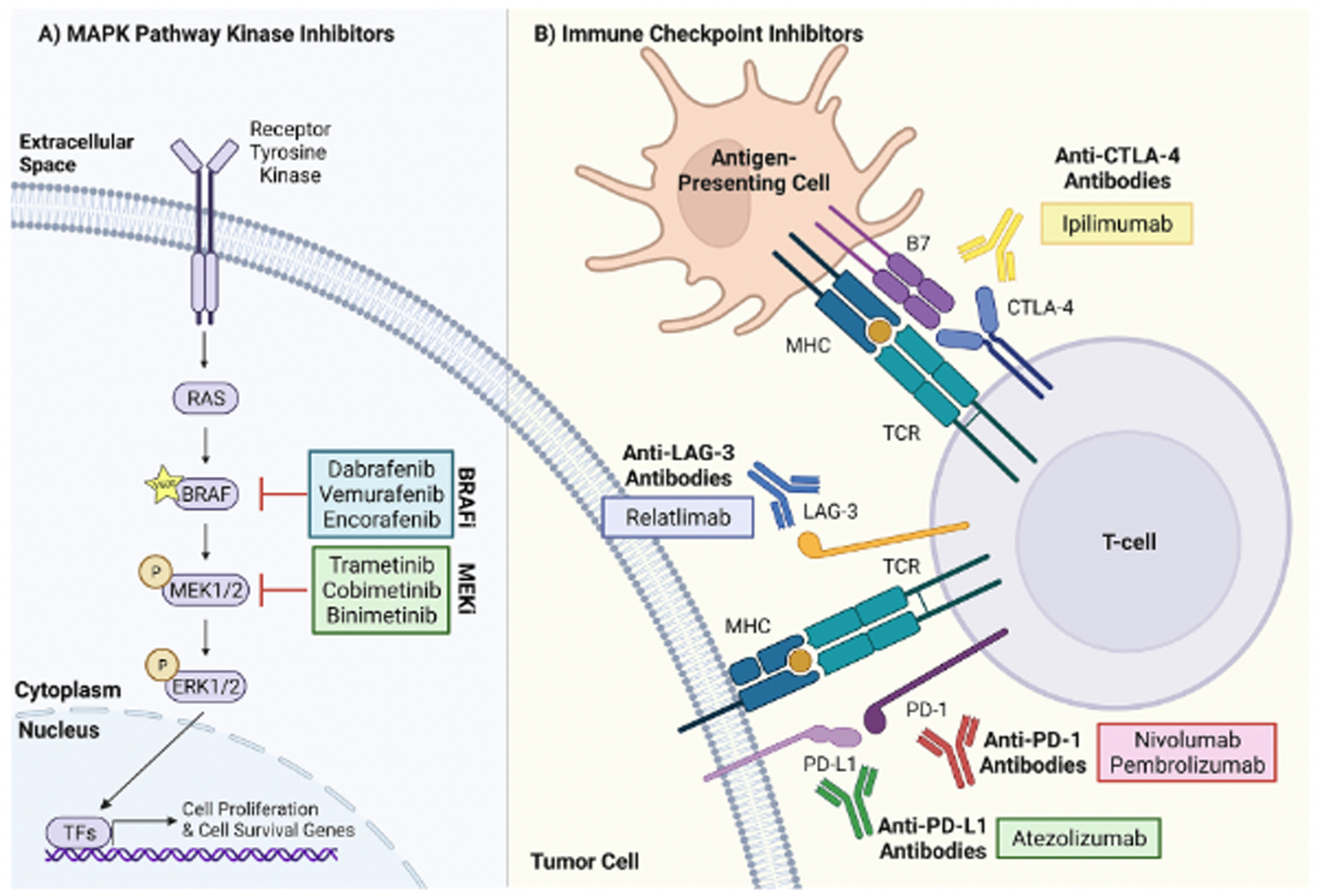
The mechanism of action of MAPK pathway kinase inhibitors and ICi approved for stage IV metastatic CM. A) V600E/K mutant BRAF drives MAPK signaling by phosphorylating (P) MEK1/2, which then phosphorylates ERK1/2. Phosphorylation occurs in the absence of RAS activation or growth factor binding to receptor tyrosine kinases upstream of BRAF. Phosphorylated ERK1/2 translocates from the cytoplasm to the nucleus, where it activates transcription factors (TFs) that promote the transcription of genes involved in cell proliferation and survival. BRAFi and MEKi inhibit V600E/K mutant BRAF and MEK1/2 activity, respectively, thereby blocking cell cycle progression and inducing cell death. B) Antigen-presenting cells and tumor cells process antigens and present them on MHC molecules, which are recognized by T-cell receptors (TCRs) on T-cells. Subsequent T-cell activation is inhibited via interactions between CTLA-4 on T-cells and B7 on antigen-presenting cells, between PD-1 on T-cells and PD-L1 on tumor cells, and between LAG-3 on T-cells and its ligands on tumor cells. Anti-CTLA-4, anti-PD-1, anti-PD-L1, and anti-LAG-3 antibodies block the inhibitory CTLA-4, PD-1, and LAG-3 checkpoints, suppressing T-cell inactivation.

Melanomas develop following the malignant transformation of melanocytes, the melanin-producing cells of the body. Melanocytes arise from neural crest cells, which originate at the neural tube, delaminate, and migrate to distal sites. Here, they express lineage-specific transcription factors and differentiate into melanocytes, amongst other cell types (Sauka-Spengler & Bronner-Fraser, 2008). Melanomas occur at various anatomical locations, and melanoma incidence, progression, and prognosis are subtype-specific (Bastian, 2014). Cutaneous melanoma of the non-acral skin (CM) is the most common subtype of melanoma, and the most fatal skin cancer (Siegel, Miller, & Jemal, 2020). Over the past 45 years, overall five-year relative survival rates for CM patients have increased and are now above 99% for patients with localized disease and up to 68% and 30% for patients with regional and distant metastases, respectively (Siegel et al., 2020). Targeted kinase inhibitors and immune checkpoint therapies have dramatically improved overall survival (OS) and progression-free survival (PFS) for patients with advanced, unresectable or metastatic CM (Fig. 1) (Curti & Faries, 2021). One phase III clinical trial recently reported a 6.5-year OS rate of 49% in patients treated with combined immune checkpoint inhibitor therapy (Wolchok et al., 2022). In contrast to CM, rare melanoma subtypes account for <10% of all melanomas and include acral lentiginous cutaneous melanoma (AM), which occurs on the palms, soles and under nails (subungual), uveal melanoma (UM) of the choroid, ciliary body, or iris, conjunctival melanoma (CJM), and mucosal melanoma (MM) which arises in the mucous membranes of the gastrointestinal tract, head and neck, and vagina (Bastian, 2014). These cancers are often diagnosed as advanced disease, are associated with poor prognosis, and have limited therapeutic options (Chacón, Pfluger, Angel, Waisberg, & Enrico, 2020).

BRAF inhibitors (BRAFi), as monotherapy or in combination with MEK inhibitors (MEKi), are approved by the U.S. Food and Drug Administration (FDA) for the treatment of metastatic CM (Curti & Faries, 2021). Both of these drug classes act on kinases in the mitogen-activated protein kinase (MAPK) pathway, which promotes cell proliferation, growth, and survival. MAPK signaling is overactive in the majority of CMs, driven by activating mutations in *BRAF* (~50%) or *NRAS* (~25%), or loss of function mutations in *NF1* (~15%) (Genomic Classification of Cutaneous Melanoma, 2015). Early clinical trials found that BRAFi (vemurafenib, dabrafenib, and encorafenib) improve median PFS as compared to chemotherapy (5.3 months with vemurafenib vs.1.6 months with dacarbazine, and 5.1 months with dabrafenib vs. 2.7 months with dacarbazine) in patients with somatic activating *BRAF* mutations (V600E or V600K substitutions), which occur in 90% of *BRAF*-mutant CMs (Chapman et al., 2011; Hauschild et al., 2012). However, the efficacy of BRAF inhibitor monotherapy is limited due to the development of drug resistance, resulting in little difference in long-term OS rates as compared to chemotherapy (4-year OS of 17% with vemurafenib vs. 16% with dacarbazine, 5-year OS of 24% with dabrafenib vs. 22% with dacarbazine) (Chapman et al., 2017; Hauschild et al., 2020). In an effort to enhance MAPK pathway inhibition and delay acquired resistance to BRAFi, combination targeted therapies consisting of BRAFi plus MEKi (dabrafenib plus trametinib, vemurafenib plus cobimetinib, encorafenib plus binimetinib), the latter of which act downstream of BRAF in the MAPK pathway, were trialed. Combination therapy improved median PFS relative to BRAF inhibitor monotherapy (9.9 months with vemurafenib plus cobimetinib vs. 6.2 months with vemurafenib, and 9.3 months with dabrafenib plus trametinib vs. 8.8 months with dabrafenib) (Larkin et al., 2014; Long et al., 2014), with the best median PFS achieved with encorafenib plus binimetinib (14.9 months vs. 9.6 months with encorafenib at median follow-up of 16.6 months) (Dummer et al., 2018).

Although most patients respond to BRAF inhibitor plus MEK inhibitor combination therapy upfront (~70% objective response rates), long-term PFS following treatment only occurs in a subset of patients (5-year PFS rates are 14% with vemurafenib plus cobimetinib, 19% with dabrafenib plus trametinib, and 22.9% with encorafenib plus binimetinib) (Ascierto et al., 2021; Dummer et al., 2021; Robert et al., 2019), and tumors often recur within months (Álamo et al., 2021). In particular, worse PFS and OS on combined BRAF inhibitor plus MEK inhibitor therapy is associated with a high baseline lactate dehydrogenase serum level and an increased number of metastatic tumor sites (Dummer et al., 2021; Hauschild et al., 2018; Saiag et al., 2021). Patients with melanoma brain metastases also show worse response to combined targeted therapy, with one phase II clinical trial reporting a 58% intracranial response rate and 6.5 months median duration of intracranial response with dabrafenib plus trametinib treatment (Davies et al., 2017). Similarly, patients with non-canonical *BRAF* mutations, which occur in 3.4% to 14% of CMs and often lead to MAPK pathway activation, experience relatively low overall response rates to BRAFi (27% and 0% for non-E/K V600 and non-V600 *BRAF* mutations, respectively), MEKi (40% for non-V600 *BRAF* mutations) and combined BRAF plus MEK inhibition (56% and 28% for non-E/K V600 and non-V600 *BRAF* mutations, respectively) (Menzer et al., 2019; Menzer & Hassel, 2022). Findings from one phase II study of patients with non-V600E/K *BRAF*-mutant tumors suggested that response to trametinib may depend on the resulting intrinsic catalytic activity of BRAF (objective response rates of 67% and 17% with high and low catalytic activity, respectively) (Nebhan et al., 2021). Furthermore, BRAFi are limited to patients with *BRAF*-mutant CM and MEKi show modest effects in *BRAF* wild-type tumors (Ascierto et al., 2013; Dummer et al., 2017; Lebbé et al., 2020; Urbonas et al., 2019) and rare melanoma subtypes (Chacón et al., 2020).

The clinical approval of immune checkpoint inhibitors (ICi) has improved outcomes for patients with metastatic CM across genetic tumor subtypes (Hughes, Klairmont, Sharfman, & Kaufman, 2021). One class of ICi, anti-cytotoxic T-lymphocyte-associated protein 4 (CTLA-4) antibodies, prevent CTLA-4 on T-cells from interacting with inhibitory signals on antigen-presenting cells, thereby increasing anti-tumor T-cell response. Treatment of CM with ipilimumab, an FDA-approved anti-CTLA-4 antibody, led to improved median OS in a phase III clinical trial (10.0 months in the ipilimumab plus glycoprotein 100 peptide vaccine arm vs. 6.4 months in the glycoprotein 100 peptide vaccine alone arm) (Hodi et al., 2010), findings that were recapitulated in a prospective phase IV trial of patients receiving ipilimumab in clinical practice (at 68.1 months median follow-up, median OS was 12.1 months) (Aamdal et al., 2022). PFS and OS in CM are further increased by nivolumab (at 60 months minimum follow-up, in the nivolumab vs. ipilimumab groups, median OS was 36.9 months vs. 19.9 months, median PFS was 6.9 months vs. 2.9 months, and objective response rates were 45% vs. 19%) (Larkin et al., 2019) and pembrolizumab (at 57.7 months median follow-up, in the pembrolizumab vs. ipilimumab groups, median OS was 32.7 months vs. 15.9 months, median PFS was 8.4 months vs. 3.4 months, and objective response rates were 42% vs. 17%) (Robert et al., 2019), ICi that target programmed death protein 1 (PD-1). Anti-PD-1 antibodies potentiate anti-tumor immunity by blocking the PD-1 receptor on T-cells from interacting with its ligands, programmed death ligand-1 (PD-L1) and programmed death ligand-2, on tumor cells or antigen-presenting cells. Combining the two classes of ICi increases patient survival more than either therapy alone (at 60 months minimum follow-up, with nivolumab plus ipilimumab, median OS was not reached, median PFS was 11.5 months, and the objective response rate was 58%) (Larkin et al., 2019), and recent clinical trials have demonstrated the efficacy of other immune checkpoint inhibitor combinations, such as nivolumab plus the anti-lymphocyte activation gene 3 (LAG-3) antibody, relatlimab, for CM treatment (Tawbi et al., 2022). Anti-CTLA4 and anti-PD-1 antibodies display more modest effects in rare melanomas (Chacón et al., 2020; D’Angelo et al., 2017).

Recently FDA-approved combination therapies, including triplet therapy with an anti-PD-L1 antibody plus vemurafenib and cobimetinib (Gutzmer et al., 2020) and doublet therapy with nivolumab plus relatlimab (Tawbi et al., 2022), will soon be entering clinical practice. However, current clinical guidelines recommend either BRAFi plus MEKi or ipilimumab plus nivolumab as first-line treatment for metastatic *BRAF*-mutant CM, while ICi are indicated for *BRAF* wild-type disease (Keilholz et al., 2020; Seth et al., 2020). BRAF inhibitor plus MEK inhibitor treatment has high response rates and leads to a rapid reduction in disease burden (Curti & Faries, 2021), whereas immune checkpoint inhibitor combination therapy has a slightly lower objective response rate (~60%), but shows a more durable response in *BRAF*-mutant CM (36% 5-year and 34% 6.5-year PFS rates with nivolumab plus ipilimumab) (Larkin et al., 2019; Wolchok et al., 2022). Retrospective cohort studies comparing first-line ipilimumab plus nivolumab vs. first-line targeted therapy also indicate that ICi lead to longer survival (Moser et al., 2019; Pavlick, Zhao, Lee, Ritchings, & Rao, 2021; Rigo et al., 2021). Thus, while the development and approval of MAPK pathway kinase inhibitors in the last decade have been a critical breakthrough for patients with CM, these therapies are being overtaken by ICi. In addition, BRAFi and MEKi lack efficacy in non-canonical *BRAF*-mutant and *BRAF* wild-type CM and in rare melanomas, further curtailing their utility. In this review, we discuss ways in which MAPK pathway kinase inhibitors are being repurposed, including in novel applications such as adjuvant and neoadjuvant therapy, in combination therapies with ICi, and in immunotherapy-resistant disease. We also highlight emerging kinase inhibitor combination therapies that are being tested in CM and in rare melanoma subtypes. Kinase inhibitors gained a foothold in melanoma treatment one decade ago and continue to play a significant role in improving outcomes for melanoma patients.

## 2. Adjuvant and neoadjuvant targeted kinase inhibitor therapy for cutaneous melanoma

Targeted kinase inhibitors were initially approved for stage IV CM; however, more recent clinical studies have focused on repurposing these drugs as adjuvant (post-surgical) and neoadjuvant (pre-surgical) therapy for earlier-stage disease (Table 1). Surgical excision is the preferred treatment for stage III melanoma, which is diagnosed in cases where the cancer has spread to nearby lymph nodes or skin lymphatic vessels with no evidence of metastasis to distant lymph nodes or organs (Michielin et al., 2020; Seth et al., 2020). The 5-year survival rates for patients with stage III CM vary widely, ranging from 93% in the lowest-stage subgroup (stage IIIA), to 32% in the highest-stage subgroup (stage IIID) (Gershenwald et al., 2017). These differences are primarily due to higher post-resection tumor recurrence rates in the most advanced cases (Romano, Scordo, Dusza, Coit, & Chapman, 2010). Adjuvant BRAF plus MEK inhibition is more effective at delaying tumor recurrence in resected stage III CM than surgical management alone, and a clinical trial is underway to minimize side effects associated with this therapy (Atkinson et al., 2021; Dummer et al., 2020). The suitability of this combination as neoadjuvant therapy is also an area of active investigation, highlighting how targeted kinase inhibitors can be adapted to new clinical applications.

### 2.1. Adjuvant targeted kinase therapy

The success of targeted kinase inhibitors and ICi for metastatic CM led to several clinical trials that investigated their efficacy in an adjuvant setting. Combining BRAFi with MEKi as adjuvant therapy improves patient outcomes, but additional studies are needed to determine which patients stand to benefit most from this approach. While adjuvant BRAF inhibitor monotherapy did not significantly increase survival in early-stage *BRAF*-mutant CM (Maio et al., 2018), adjuvant dabrafenib plus trametinib combination therapy for stage III V600 *BRAF*-mutant CM was approved by the FDA in 2018 based on results from the COMBI-AD trial. Patients randomized to dabrafenib plus trametinib vs. placebo showed higher 3-year (59% vs. 40%) and 4-year (54% vs. 38%) recurrence-free survival rates and better distant metastasis-free survival, irrespective of baseline clinical factors (Hauschild et al., 2018; Long et al., 2017). At 5-year follow-up, 52% of patients treated with adjuvant targeted therapy had not experienced relapse, compared to 36% in the placebo group (Dummer, Hauschild, et al., 2020). Patients across stage III subgroups saw improvements in recurrence-free survival, and these findings have held up in retrospective analyses of clinical practice (Rauwerdink et al., 2020; Torphy et al., 2022). Pyrexia was the most common adverse event reported with treatment in the COMBI-AD trial, as well as the most common reason for discontinuation of the adjuvant therapy (Atkinson et al., 2021; Long et al., 2017). Preliminary data from the COMBI-APlus trial (NCT03551626), which is evaluating the impact of a novel clinical management protocol on pyrexia-related adverse events, shows improved tolerability to the therapy (Atkinson et al., 2021). Other ongoing studies with this combination are observational, examining treatment administration and adherence in the clinic (NCT03944356), assessing the quality of life for patients on the treatment (NCT04547946), and determining response rates in real-world clinical practice (NCT04961619, NCT05171374, NCT04666272). Results from these studies will demonstrate the feasibility and efficacy of adjuvant BRAFi and MEKi in a clinical setting, as opposed to a clinical trial.

Several open questions for targeted kinase inhibitors as adjuvant therapy remain. There have been no trials directly comparing adjuvant immunotherapy vs. dabrafenib plus trametinib for resectable *BRAF*-mutant CM, and predictive markers for both therapies are an area of active investigation (Tonella et al., 2021). Thus, the choice of which adjuvant treatment to pursue is left up to the clinician and patient (Michielin et al., 2020; Seth et al., 2020). Additionally, it is unclear if adjuvant BRAFi plus MEKi should be used for stage IIIA tumors. The COMBI-AD trial enrolled patients based on the American Joint Committee on Cancer (AJCC) 7th edition staging criteria, which did not include the stage IIID subgroup that has since been defined in the AJCC 8th edition staging criteria, thereby negatively skewing prognosis for stage IIIA patients (Gershenwald et al., 2017). Post hoc analysis of recurrence-free survival using an AJCC 8th edition staging framework demonstrated less benefit with adjuvant dabrafenib plus trametinib for patients with stage IIIA disease, such that the use of this therapy needs to be carefully weighed against potential side effects (Christofyllakis et al., 2020; Hauschild, Dummer, et al., 2018; Michielin et al., 2020). Additionally, the COMBI-AD inclusion criteria required that patients with stage IIIA disease have lymph node metastasis of >1 mm, and so the efficacy of adjuvant dabrafenib plus trametinib in patients with limited nodal involvement remains unclear (Long et al., 2017). A limitation to adjuvant BRAF plus MEK inhibitor therapy is that *BRAF* wild-type patients are not eligible. Instead, adjuvant nivolumab or pembrolizumab monotherapy can be used irrespective of *BRAF* mutational status. These adjuvant therapies demonstrated improvements in recurrence-free survival and are now FDA-approved for stage III CM (Christofyllakis et al., 2020). Adjuvant pembrolizumab was approved in late 2021 for non-metastatic (stage II) CM following favorable results from the KEYNOTE-716 trial (NCT03553836), and additional trials are assessing the efficacy of nivolumab for these patients (NCT04309409, NCT04099251, NCT03405155). Furthermore, the CheckMate 238 study led to the approval of adjuvant nivolumab in resected stage IIIB/C or IV CM (Ascierto et al., 2020). Conversely, the application of adjuvant BRAFi plus MEKi for high-risk stage II and resected metastatic stage IV CM has yet to be evaluated in prospective clinical trials. In fact, the COLUMBUS-AD study (NCT05270044/EORTC-2139-MG), a randomized triple-blind placebo-controlled phase III clinical trial, will be the first to examine the efficacy of adjuvant BRAF inhibitor plus MEK inhibitor (12-months of encorafenib plus binimetinib) for patients with high-risk stage II CM. The results from COMBI-AD suggest this could be a promising therapy for *BRAF*-mutant tumors if side effects are appropriately managed.

### 2.2. Neoadjuvant targeted kinase therapy

Several phase I/II trials are examining neoadjuvant therapy for resectable early-stage CM. Initial findings for neoadjuvant BRAF plus MEK inhibitor therapy are encouraging, showing tolerability, delayed tumor recurrence, and complete pathological responses. Assessing patients for pathological response via evaluation of the excised tumor specimen after neoadjuvant therapy is particularly important given that complete pathological response is correlated with improved recurrence-free survival and OS (Menzies et al., 2021). However, the durability of response with neoadjuvant BRAFi plus MEKi is limited, and additional trials testing various combinations of targeted therapies with or without ICi in a neoadjuvant setting have been initiated. In a small phase II trial, stage III or oligometastatic stage IV *BRAF*-mutant CM treated with 8-weeks of neoadjuvant dabrafenib plus trametinib and adjuvant therapy showed significant improvement in event-free survival as compared to the standard of care arm, and 58% of patients on neoadjuvant therapy had complete pathological response (Amaria et al., 2018). In the NeoCombi trial, 12-weeks of neoadjuvant dabrafenib plus trametinib in stage III disease resulted in almost half of all patients (49%) showing complete pathological response (Long et al., 2019), findings that were recapitulated in a retrospective analysis of stage III and IV patients treated with neoadjuvant targeted kinase inhibitors (Eroglu et al., 2020). In addition to improving post-operative survival, neoadjuvant dabrafenib plus trametinib has been effectively used as cytoreductive therapy, enabling surgical resection of previously unresectable tumors (Blankenstein et al., 2021).

Neoadjuvant ICi also demonstrate high pathological response rates and improvements in survival for early-stage CM. However, immune checkpoint inhibitor therapy can be accompanied by acute toxicity, as well as chronic and rarely fatal immune-related adverse events (Amaria et al., 2018; Blank et al., 2018; Johnson, Nebhan, Moslehi, & Balko, 2022), whereas the toxicities reported with BRAF and MEK inhibitor combination therapy lead to frequent discontinuation of treatment but are moderate and reversible (Heinzerling et al., 2019). Neoadjuvant dabrafenib plus trametinib showed toxicity similar to that seen in an adjuvant treatment setting, suggesting that BRAFi plus MEKi could be appropriate as neoadjuvant therapy for patients at risk of adverse events with ICi (Amaria, Prieto, et al., 2018; Long et al., 2019). Unfortunately, results from the NeoCombi trial showed that patients treated with neoadjuvant dabrafenib plus trametinib still experience tumor progression, regardless of their pathological response (Long et al., 2019). The 1-year and 2-year relapse-free survival rates across pathological responses were 77.1% and 43.4%, respectively. A pooled analysis comparing neoadjuvant anti-PD-1-based immunotherapy vs. neoadjuvant dabrafenib plus trametinib showed similar pathological response rates and survival outcomes across the two treatment approaches; however, immunotherapy confers improved recurrence-free survival (78% vs. 75% 1-year, and 75% vs. 47% 2-year) (Menzies et al., 2021). Efforts are underway to determine the efficacy of alternative neoadjuvant BRAF and MEK inhibitor combinations in stage III and stage IV CM, including vemurafenib plus cobimetinib (NCT02036086) and encorafenib plus binimetinib (NCT04741997, NCT04221438, NCT05097378). Additionally, neoadjuvant targeted therapy is being tested in combination with anti-PD-1 and anti-PD-L1 antibodies, including neoadjuvant pembrolizumab (NCT02858921) and atezolizumab (NCT03554083, NCT04722575), or prior to adjuvant spartalizumab (NCT04310397). Although the use of neoadjuvant therapy in CM is still emerging, targeted kinase inhibitors could be important for a subset of patients with *BRAF*-mutant tumors.

## 3. Targeted kinase inhibitor and immunotherapy combinations for cutaneous melanoma

The efficacy of ICi depends on the tumor immune microenvironment (TIME) and the immunogenicity of tumor cells. Predictors of strong clinical response include higher density and tumor infiltration of cytotoxic CD8+ T-cells, higher expression of PD-L1 and major histocompatibility complex (MHC) molecules on tumor cells, increased tumor mutational burden or microsatellite instability, and enriched immune-related gene signatures (Giraldo et al., 2019; Rizk et al., 2020). Pre-clinical studies have demonstrated that BRAFi create a pro-inflammatory TIME by increasing the abundance and activity of cytotoxic CD8+ and helper CD4+ tumor-infiltrating lymphocytes (TILs) (Cooper et al., 2014; Erkes et al., 2020; Ho et al., 2014), increasing infiltration and proliferation of natural killer cells (Ferrari de Andrade et al., 2014; Frazao et al., 2017), and reducing the relative accumulation of regulatory T-cells (Ho et al., 2014; Steinberg et al., 2014). Furthermore, BRAFi downregulate immunosuppressive cytokines (Liu et al., 2015; Ott et al., 2013) and restore the expression of MHC molecules on tumor cells, which reduces immune evasion (Bradley et al., 2015; Sapkota, Hill, & Pollack, 2013). Tumor recognition by T-cells is further enhanced by BRAFi via increased expression of melanoma antigens on cancer cells (Boni et al., 2010; Pieper et al., 2018). The immune-modulatory effects of MEKi are mixed, leading to decreased T-cell proliferation, activity, and priming, while also increasing the accumulation and survival of CD8+ TILs, enhancing MHC and melanoma antigen expression, and downregulating immunosuppressive cytokines (Boni et al., 2010; Ebert et al., 2016; Liu et al., 2015; Vella et al., 2014). Adding MEKi to BRAFi also increases CD8+ and CD4+ TILs (Homet Moreno, Mok, Comin-Anduix, Hu-Lieskovan, & Ribas, 2016); anti-tumor responses mediated by this combination are T-cell dependent (Erkes et al., 2020). These pre-clinical findings align with the increase in TILs and melanoma antigens seen in tumor samples from patients treated with BRAFi and/or MEKi (Cooper et al., 2014; Deken et al., 2016; Frederick et al., 2013; Wilmott et al., 2012). The pro-immunogenic effects of BRAFi and MEKi on the TIME provide a strong rationale for coupling them with ICi.

### 3.1. Concurrent targeted kinase and immune checkpoint inhibitor combinations

Several clinical trials are examining strategies for administering BRAFi and MEKi in combination with ICi (Table 2). PD-1 blockade enhances the effects of MAPK pathway targeting agents *in vivo* (Cooper et al., 2014; Deken et al., 2016; Ebert et al., 2016; Hu-Lieskovan et al., 2015; Liu et al., 2015), and these findings are emulated in clinical trials where ICi are administered concurrently with BRAFi and MEKi. Dual immune checkpoint inhibition with combined targeted kinase inhibition may be unfavorable due to the increased risk of side effects with a four-drug regimen (Daud & Tsai, 2017; Haanen et al., 2017), and the safety and tolerability of this approach is being tested in the phase I/II QUAD01 study (NCT04655157), which is treating unresectable or metastatic CM patients with a combination of encorafenib, binimetinib, nivolumab, and ipilimumab. The majority of clinical trials have instead focused on doublet or triplet combination therapy. Ipilimumab in combination with vemurafenib or dabrafenib plus trametinib resulted in severe toxicity (Minor, Puzanov, Callahan, Hug, & Hoos, 2015; Ribas, Hodi, Callahan, Konto, & Wolchok, 2013); however, concurrent combination therapies with anti-PD-1 and anti-PD-L1 antibodies have been more promising. The KEYNOTE-022 phase II study of pembrolizumab plus dabrafenib and trametinib showed higher PFS with the triplet therapy as compared to the targeted therapy combination alone, with statistical significance reached after a longer median follow-up of 36.6 months (Ascierto et al., 2019; Ferrucci et al., 2020). Conversely, findings from COMBI-i, which tested spartalizumab in combination with dabrafenib plus trametinib demonstrated no improvement in PFS with triplet therapy compared to targeted therapy alone (Dummer et al., 2022). A phase I trial of durvalumab, an anti-PD-L1 antibody, in combination with dabrafenib plus trametinib led to overall response rates similar to those historically seen with targeted therapy, although toxicities were increased with the combination (Ribas et al., 2020). In contrast, in the IMspire150 study, a regimen utilizing atezolizumab, another anti-PD-L1 antibody, in combination with vemurafenib plus cobimetinib, demonstrated increased PFS over targeted therapy alone (15.1 months vs.10.6 months at median follow-up of 18.9 months) in *BRAF* V600-mutant CM (Gutzmer et al., 2020). The tolerable safety profile of this triplet therapy led to the FDA approval of this combination for *BRAF*-mutant CM in 2020.

Additional trials examining concurrent therapies with ICi and BRAFi/MEKi are ongoing. Triplet combination therapy consisting of pembrolizumab plus encorafenib and binimetinib was found to be tolerable and is being studied in a phase III trial (NCT04657991) (Zimmer et al., 2021). Preliminary data from the TRIDENT trial (NCT02910700) of nivolumab combined with dabrafenib and trametinib shows high objective response rates in patients that are naïve (100%) or refractory (88%) to anti-PD-1 therapy and in patients with melanoma brain metastases (57%) (Burton et al., 2021). Many patients experienced side effects in this study, including 78% of patients with grade 3/4 treatment-related adverse events, and 22% of patients discontinued treatment (Burton et al., 2021). Future investigations will need to evaluate whether the toxicity associated with concurrent combination therapy is appropriate, given potentially marginal improvements in response and survival (Liu, Zhang, Wang, & Cui, 2021). Nonetheless, the approval of the triplet combination used in the IMspire150 trial is encouraging; future studies could consider concurrent treatment with next-generation ICi, such as anti-LAG-3 antibodies, to further boost the effectiveness of BRAFi and MEKi.

### 3.2. Sequential targeted kinase and immune checkpoint inhibitor combinations

Toxicity and tolerability have been major concerns for concurrent BRAF, MEK, and immune checkpoint inhibitor triple therapy. Efforts are underway to understand whether intermittent dosing or sequential scheduling of these therapies could minimize side effects while improving survival. This is a delicate area of investigation given that intermittent dosing of BRAF plus MEK inhibitor therapy following disease stabilization does not improve outcomes compared to continuous dosing (Algazi et al., 2020; Gonzalez-Cao et al., 2021). The IMPemBra trial (NCT02625337) is comparing pembrolizumab monotherapy vs. pembrolizumab with intermittent or continuous dabrafenib plus trametinib. Preliminary findings suggest that the intermittent use of BRAF plus MEK inhibitor improves median PFS over continuous pembrolizumab monotherapy (27.0 months vs. 10.6 months at 17.4 months median follow-up). This regimen was also better tolerated than concurrent triple therapy, although adverse events including pyrexia and liver toxicity still led to discontinuation of treatment (Rozeman et al., 2020). Early results from parts 4 and 5 of the KEYNOTE-022 trial, which is investigating pembrolizumab plus intermittent or concurrent dosing of trametinib in *BRAF* wild-type tumors, show numerically higher response rates with intermittent dosing, although responses are low in both conditions (Maio et al., 2022). Longer follow-up will help determine whether intermittent targeted therapy plus immune checkpoint inhibition is superior to concurrent triplet therapy.

Trials examining the sequencing of targeted therapy and immunotherapy offer an opportunity for a head-to-head comparison between the two treatments, while examining whether progression on one therapy impedes response to the other. The safety and feasibility of a scheduling approach was demonstrated early on with vemurafenib followed by ipilimumab (Amin et al., 2016), and with durvalumab followed by trametinib in *BRAF* wild-type CM (Ribas et al., 2020), and showed less toxicity than concurrent administration of the drugs. Preliminary data have recently been reported from the DREAMseq study (NCT02224781), which randomized patients to dabrafenib plus trametinib followed by crossover to ipilimumab plus nivolumab upon disease progression, or the inverse sequence. At a median follow-up of 27.7 months, 27 patients had switched from ipilimumab plus nivolumab to targeted therapy and 46 from targeted therapy to ipilimumab plus nivolumab (Atkins et al., 2021). Although toxicity was slightly increased in the ICi-first arm, objective response rates were higher than in the targeted therapy-first arm (46% vs. 43%) and were more sustained following treatment cross-over (48% vs. 30%). In line with objective response rates, the 2-year OS for patients starting on ipilimumab plus nivolumab was higher than for those starting on targeted therapy, with PFS following a similar trend (Atkins et al., 2021). Preliminary findings from the SECOMBIT trial (NCT02631447) support this result. This study randomized *BRAF*-mutant metastatic CM patients into three arms: encorafenib plus binimetinib followed by crossover to ipilimumab plus nivolumab upon disease progression, the inverse sequence, or a sandwich approach where induction with 8-weeks of encorafenib plus binimetinib was followed by ipilimumab plus nivolumab until disease progression, at which point targeted therapy was initiated again. OS and PFS rates at 2- and 3-years follow-up were higher in the ICi-first and sandwich scheduling arms as compared to the targeted therapy-first approach (Ascierto et al., 2021). These studies suggest that initiating ICi before disease progression on BRAFi and MEKi is preferred, corroborating *in vitro* and clinical observations that showed acquired resistance to targeted therapy is associated with an immunosuppressed TIME (Cooper et al., 2016; Frederick et al., 2013; Hugo et al., 2015; Smith et al., 2014; Steinberg et al., 2017) and cross-resistance to ICi (Haas et al., 2021; Lau et al., 2021).

### 3.3. Induction therapy with targeted kinase inhibitors

Although immune checkpoint blockade provides a more durable clinical response than targeted therapy, 40–65% of patients show no initial response and almost half of all patients develop acquired resistance and progress within 3 years (Gide, Wilmott, Scolyer, & Long, 2018). BRAFi and MEKi boost anti-tumor immunity, and these effects appear to be relatively acute, suggesting these drugs could be used to prime the immune system prior to initiating ICi. CM cells treated with BRAFi for 3 or 7 days, but not 14 or 21 days, activated CD8+ T-cells *in vitro* via upregulation of melanoma antigens (Pieper et al., 2018). Patient samples taken early during targeted therapy treatment (<1 week up to 1 month) show high TIL counts that are absent at the time of disease progression (Kakavand et al., 2015; Wilmott et al., 2012). One study found decreased TILs in biopsies taken more than 15 days after starting targeted therapy treatment (Deken et al., 2016). In pre-clinical models, lead-in treatment with trametinib followed by anti-PD-1 plus trametinib treatment improved survival more than the same therapy with anti-PD-1 lead-in (Liu et al., 2015).

Several clinical trials are attempting to leverage the critical window of therapeutic opportunity for targeted inhibitor lead-in that occurs well before disease progression. Importantly, the approval of atezolizumab in combination with vemurafenib plus cobimetinib followed data from the IMspire150 study, which included a 28-day induction period with vemurafenib plus cobimetinib prior to beginning the triplet therapy (Gutzmer et al., 2020). Further clinical evidence that lead-in with targeted therapy could work comes from the sandwich scheduling arm of SECOMBIT, which shows similar PFS and OS rates to the ICi-first sequential approach (Ascierto, Mandala, et al., 2021). The ImmunoCobiVem trial (NCT02902029) starts with 3 months of vemurafenib plus cobimetinib as induction therapy, followed by randomization to either vemurafenib plus cobimetinib and crossover to atezolizumab upon disease progression, or the inverse sequence. The EBIN study (NCT03235245) is examining the efficacy of a 12-week induction period with encorafenib plus binimetinib followed by ipilimumab plus nivolumab, vs. the use of ipilimumab plus nivolumab alone. Similarly, COWBOY (NCT02968303) is studying the efficacy of 6-weeks induction therapy with vemurafenib plus cobimetinib, followed by ipilimumab plus nivolumab, vs. upfront ipilimumab plus nivolumab. Although the optimal length of induction therapy is unclear, and the decision of when to begin ICi in a clinical setting could be complicated by the lack of early biomarkers for acquired resistance to BRAFi and MEKi (Tarhini & Kudchadkar, 2018), these trials are beginning to evaluate whether lead-in targeted therapy can delay progression on ICi. Intriguingly, *in vivo* pre-clinical studies of lead-in anti-PD-1 and anti-PD-L1 therapy before immune checkpoint plus BRAF/MEK inhibitor combination therapy were more effective than concurrent therapy without lead-in (Y. Wang et al., 2021), introducing another induction approach that should be tested clinically. Thus, although the optimal sequencing of ICi and BRAFi/MEKi still needs further examination, it appears that targeted kinase inhibitors can successfully complement ICi.

### 3.4. Beyond checkpoint inhibitor immunotherapies

Although ICi are the immunotherapy of choice for CM, several other immune-modulating treatments are either already approved or are being actively developed for CM treatment, including oncolytic virus therapy, cytokine treatment, and adoptive cell transfer. Much like with ICi, clinical trials combining these alternative immunotherapies with targeted kinase inhibitors are testing the ability of BRAFi and MEKi to enhance their anti-tumor effects. The FDA approved the use of talimogene laherparepvec (T-VEC), an oncolytic virus therapy, for the local treatment of unresectable recurrent CM in 2015 following results from the OPTiM trial (Andtbacka et al., 2015). T-VEC is a modified herpes simplex virus 1 that, once injected into a tumor, infects cancer cells, leading to cell lysis and subsequently boosting immune responses (Ferrucci, Pala, Conforti, & Cocorocchio, 2021). In addition to exerting local cytotoxic effects, T-VEC has been proposed to have systemic antitumor effects beyond the injection site. Results from the OPTiM trial showed that uninjected CM lesions regressed after T-VEC administration, albeit to a lesser extent than injected lesions. Pre-clinical *in vivo* studies across cancer types, including CM, have shown that combining oncolytic herpes simplex virus with BRAFi or MEKi delays tumor growth more than either treatment alone (Bommareddy, Aspromonte, Zloza, Rabkin, & Kaufman, 2018; Crespo-Rodriguez et al., 2020; Zhou et al., 2021). These clinical and pre-clinical findings have led to the initiation of a trial that is testing the safety and tolerability of combining T-VEC with dabrafenib plus trametinib (NCT03088176) in *BRAF*-mutant CM. The efficacy of this combination is of particular interest given that combining pembrolizumab with T-VEC in the phase III MASTERKEY-265 trial did not lead to a statistically significant improvement in PFS or OS for patients with unresectable or metastatic CM (Ribas et al., 2021).

Cytokine immunotherapies have also been studied in combination with BRAFi and MEKi in CM. High-dose interleukin-2 (HD-IL-2) stimulates T-cells but has limited efficacy as monotherapy (Alva et al., 2016) or in combination with vemurafenib (Clark et al., 2018; Mooradian et al., 2018). Interferon-alpha-2b (IFN) also stimulates immune responses and was approved as an adjuvant therapy for early-stage CM in 2011 (Mocellin, Pasquali, Rossi, & Nitti, 2010). Pre-clinical studies show that BRAFi increase the expression of the IFNAR1 receptor on *BRAF*-mutant tumor cells, potentiating the activity of IFN (Sabbatino et al., 2016), and a phase I study combining IFN with vemurafenib plus cobimetinib demonstrated the safety of this triple therapy in the clinic (Simeone et al., 2021). A larger-scale clinical trial would be needed to demonstrate the impact of this combination on patient survival, although the utility of such a study is unclear given that cytokine immunotherapies are no longer recommended for routine clinical practice (Seth et al., 2020). Adoptive cell therapy (ACT) with TILs, which involves the retrieval of TILs from resected patient tumors, their expansion *ex vivo*, and infusion back into patients following lymphodepletion with chemotherapy, is also effective in CM (Rohaan, van den Berg, Kvistborg, & Haanen, 2018), including in patients refractory to targeted therapies or ICi (Sarnaik et al., 2021). BRAFi and/or MEKi sensitize tumors to ACT with TILs in pre-clinical and pilot clinical studies (Deniger et al., 2017; Hu-Lieskovan et al., 2015; Koya et al., 2012) and should be considered in combination with ACT as this therapeutic strategy continues to evolve in the future. As with ICi, targeted kinase inhibitors could play a key role in potentiating alternative immunotherapies.

## 4. New kinase inhibitor therapies and combinations for cutaneous melanoma

**Fig. 2.**
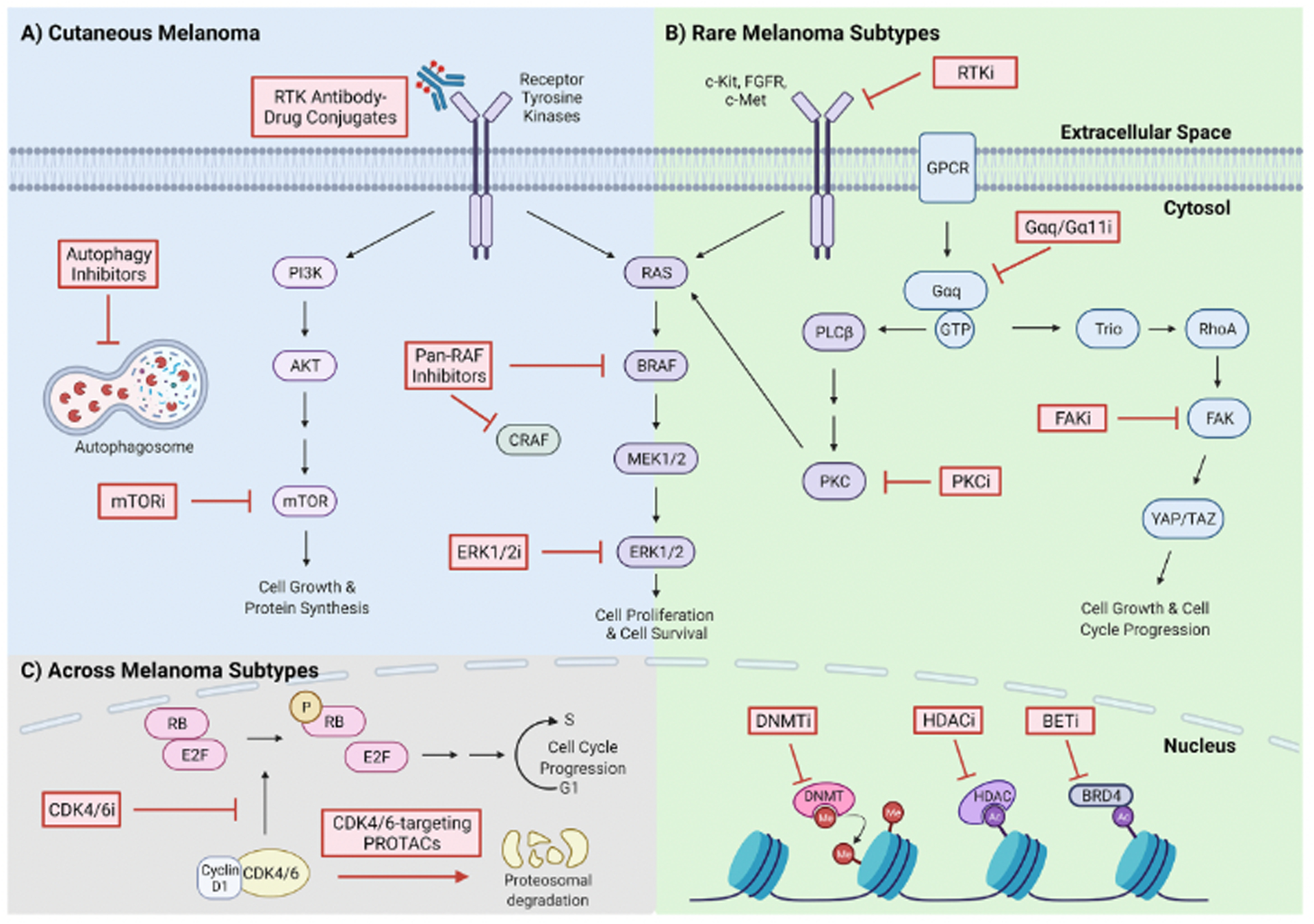
Selected actionable drug targets, for monotherapy or in combination with BRAFi and/or MEKi, in CM and rare melanoma subtypes. A) Combination therapies for CM. RTKs, which activate both the MAPK and PI3K/AKT/mTOR survival pathways, can be inhibited by RTK-targeting antibody-drug conjugates. mTORi act downstream of RTKs on the PI3K/AKT/mTOR pathway. Pan-RAF inhibitors, which prevent RAF monomer and/or dimer activity, ERK1/2i, which prevent ERK1/2 phosphorylation, and autophagy inhibitors, which prevent autophagosome maturation, are also being tested in combination with BRAFi and/or MEKi. B) Combination therapies for rare melanoma subtypes. RTKs activate MAPK signaling in rare melanomas and can be targeted by small-molecule RTK inhibitors (RTKi), such as cMET or FGFR inhibitors in UM and c-Kit inhibitors in AM and MM. Active G-protein signaling contributes to MAPK pathway signaling and cell survival in UM via PLCß/PKC. Targeted inhibitors of Gαq/11 and PKC can be combined with MEKi for UM. In parallel, Trio/RhoA/FAK/YAP pathway activation leads to increased cell growth and cell cycle progression and can be targeted with FAKi. Epigenetic modifications, which are common in UM, can be targeted with DNA methyltransferase inhibitors (DNMTi), HDACi, and BETi. C) Combination therapies across melanoma subtypes. Cell cycle dysregulation in melanoma is driven by cyclin D1-CDK4/6 complex phosphorylation (P) of retinoblastoma protein (RB), and the subsequent release of the E2F transcription factor, which drives G1 to S phase cell cycle progression. CDK4/6i and CDK4/6-targeting PROTACs block cell cycle progression by preventing the kinase activity of CDK4/6 or by destining CDK4/6 for proteolytic degradation, respectively, and can be used in combination with MEKi.

BRAF and MEK inhibitors have made considerable strides in improving outcomes for CM patients. However, the development of drug resistance limits their durability (Czarnecka, Bartnik, Fiedorowicz, & Rutkowski, 2020). Resistance to targeted therapy can be mediated by MAPK pathway reactivation (Sanchez et al., 2019; Van Allen et al., 2014; Wagle et al., 2014), the induction of alternative survival pathways (Catalanotti et al., 2017; Shi et al., 2014; Shi et al., 2014; Van Allen et al., 2014; B. Wang et al., 2021), and increased expression of receptor tyrosine kinases (RTKs) (Abel et al., 2013; Nazarian et al., 2010; Patel et al., 2021) or cell cycle proteins (Smalley et al., 2008). Metabolic adaptations (Avagliano et al., 2020) and increased tolerance to oxidative stress (Khamari et al., 2018; Nguyen et al., 2020) have also been implicated in resistance. Furthermore, the tumor microenvironment influences both signaling and metabolic pathways to mediate BRAF/MEK inhibitor resistance (Falcone et al., 2020), via cancer cell interactions with immune cells (Erkes et al., 2020; Tabolacci et al., 2021), cancer-associated fibroblasts (Alicea et al., 2020; Capparelli, Rosenbaum, Berger, & Aplin, 2015; Straussman et al., 2012), or extracellular matrix components (Berestjuk et al., 2022; Fedorenko et al., 2016). Many of these mechanisms are associated with phenotype switching to a drug-tolerant cell state via transient changes in transcriptional programs before the acquisition of stable drug resistance (Arozarena & Wellbrock, 2019; Shaffer et al., 2017). Developing therapies against these drivers of drug resistance can delay tumor recurrence, and a sampling of novel targeted inhibitors is being tested in combination with BRAFi and MEKi for the treatment of CM (Fig. 2).

### 4.1. MAPK pathway kinases

One therapeutic approach that can prevent or delay progression on targeted therapy is the inhibition of kinases vertically downstream or upstream of BRAF and MEK. Downstream of MEK, increased ERK1/2 kinase activity leads to acquired drug resistance (Morris et al., 2013; Sanchez et al., 2019; Wagle et al., 2014); however, ERK1/2 inhibitors (ERK1/2i) have shown limited clinical activity as single agents in melanoma, including in a patient with non-E/K V600 *BRAF* mutant CM, and in other solid tumors (Janku et al., 2020; Moschos et al., 2018; Sullivan et al., 2018). Ongoing trials are testing the safety and efficacy of ERK1/2i as single agents (NCT04198818, NCT04488003), or in combination with other therapies (NCT02857270, NCT02972034, NCT03745989, NCT02457793), including BRAFi, MEKi, and ICi, across MAPK-mutant cancers. Upstream of BRAF, activating *NRAS* mutations (most frequently at the Q61 codon) are mediators of intrinsic targeted therapy resistance in CM, and activation of wild-type KRAS leads to acquired resistance (Dietrich, Kuphal, Spruss, Hellerbrand, & Bosserhoff, 2018). RAS GTPases were considered ‘undruggable’ until recently, when sotorasib produced clinical responses in solid tumors with *KRAS*^*G12C*^ mutations (Hong et al., 2020). RAS inhibitors for CM still need to be developed and would ideally be *NRAS*^*Q61R/K/L*^ mutation specific. Combining RAS inhibitors with targeted kinase inhibitors against MEK, ERK1/2, or upstream activators of mutant NRAS, could further maximize therapy response.

RTKs are transmembrane receptors that undergo auto-phosphorylation upon ligand binding and activate downstream survival pathways, including the MAPK pathway. Upregulation and activation of RTKs is a well-described mechanism of acquired resistance to BRAFi and MEKi that can be targeted with small molecule inhibitors or anti-RTK antibodies (Sabbah et al., 2021). In particular, pre-clinical studies have identified autocrine and paracrine activation of EGFR, IGFR, PDGFR-alpha, PDGFR-beta, c-MET, FGFR, and AXL as drivers of targeted therapy resistance (Müller et al., 2014; Nazarian et al., 2010; Patel et al., 2021; Sabbatino et al., 2014; Straussman et al., 2012; Sun et al., 2014; Wang et al., 2019). Early clinical trials tested RTK inhibitors as monotherapy, matching treatments to a tumor’s genetic profile, but showed low response rates in CM (Sabbah et al., 2021). Current efforts are examining the safety and efficacy of combining selective FGFR or cMET inhibitors with BRAFi plus MEKi (NCT02159066) and testing novel AXL inhibitors in solid tumors, including CMs (NCT02729298, NCT04458259). Increasingly, pan-RTK inhibitors that bind multiple RTKs are being considered across treatment modalities, including as monotherapy (NCT01831726, NCT02571036, NCT04771520) for solid tumors, in combination with ICi for advanced CM (NCT03957551, NCT04493203, NCT04091750), or as a neoadjuvant for stage III disease (NCT04207086). Theoretically, multi-targeting RTK inhibitors remain personalized therapies that act against tumor-specific mutations, but they also have the potential to be effective against heterogeneous tumors that display multiple mechanisms of tumorigenesis or drug resistance.

Monoclonal antibodies can also be used to target overactive RTKs. For example, blocking ERBB3/HER3 and ERBB2/HER2 signaling in resistant *BRAF*-mutant and *BRAF* wild-type tumors with anti-ERBB3 or anti-ERBB2 antibodies lowers MAPK pathway signaling and increases sensitivity to BRAFi and MEKi (Capparelli et al., 2015; Capparelli et al., 2018). Furthermore, anti-RTK antibodies can be developed as antibody-drug conjugates (ADCs). These biologics contain a monoclonal antibody that recognizes a cancer cell target surface receptor or antigen linked to a cytotoxic drug, which gets delivered to cancer cells once the ADC is internalized, thereby sparing normal tissue (Drago, Modi, & Chandarlapaty, 2021). Two ADCs are FDA-approved for HER2-positive breast cancer: trastuzumab emtansine and trastuzumab deruxtecan. An ADC comprised of an AXL antibody conjugated to an antimitotic drug blocked tumor growth *in vitro* and *in vivo* in treatment-naïve and targeted therapy-resistant CM, and these effects were enhanced upon the addition of BRAFi and MEKi (Boshuizen et al., 2018). Notably, the AXL antibody had no anti-tumor activity on its own, requiring the cytotoxic drug to exert an effect. Several clinical trials examining the efficacy of ADCs in CM have been initiated, including RTK-targeting conjugates against AXL (NCT02988817) and ERBB2/HER2 (NCT05135715). Combination therapy with ADCs plus BRAFi and/or MEKi should continue to be explored in CM given that it has the potential to delay tumor recurrence and is also relatively selective for tumor cells; this can minimize toxicity, which otherwise often restricts the use of combination therapies.

Constitutive RAS activation in *NRAS*-mutant CMs, and alternative splicing of BRAF V600E in the absence of RAS activity, promote RAF dimerization, which drives tumor progression and resistance to BRAFi (Brummer & McInnes, 2020). BRAFi exert their anti-tumor effects by targeting the monomeric form of V600-mutant BRAF. Paradoxically, the addition of BRAFi to CMs with RAS activation or alternatively spliced BRAF-V600E isoforms enhances RAF dimerization and hyperactivates MAPK signaling (Poulikakos et al., 2011; Poulikakos, Zhang, Bollag, Shokat, & Rosen, 2010). This discovery led to the development of paradox-breaking BRAFi, which prevent RAF dimerization (BRAF homodimers and BRAF-CRAF heterodimers) and limit the growth of cells that are *BRAF* wild-type or have alternatively spliced BRAF (Hartsough et al., 2018; Yao et al., 2019). Preliminary data from a phase I/II study of PLX8394, a paradox-breaking BRAFi, led to stable disease in 30% of refractory solid tumors (Janku et al., 2018), and recruitment is ongoing (NCT02428712).

There has also been renewed interest in pan-RAF inhibitors, which target both BRAF and CRAF and can inhibit the kinase activity of RAF monomers and/or dimers. Pan-RAF inhibitors induce cell death in BRAF/MEK inhibitor-naïve and -resistant cell lines (Brummer & McInnes, 2020; Hong et al., 2021), and enhance the activity of MEKi in non-V600 *BRAF* mutant CMs (Atefi et al., 2015; Hong, Piva, et al., 2021; Molnár et al., 2018; Shi et al., 2012). Phase I studies with pan-RAF inhibitor monotherapy have thus far been disappointing, showing minimal-to-no response in *BRAF*-mutant and *NRAS*-mutant CMs (Dickson et al., 2015; Hong et al., 2016; Kim et al., 2019; Olszanski et al., 2017; Sullivan et al., 2020). Results from additional trials with pan-RAF inhibitor monotherapy (NCT02437227, NCT02607813, NCT04985604) are pending. Conversely, treatment with lifirafenib (BGB-283), a RAF dimer and EGFR kinase inhibitor, led to complete and partial responses in patients with *BRAF*-mutant CM, suggesting that multiple nodes in the MAPK pathway may need to be targeted for this therapy to be effective (Desai et al., 2020). Pan-RAF inhibitors in combination with MEKi, ERK1/2i, or cyclin-dependent kinase-4/6 inhibitors (CDK4/6i), are currently being studied in clinical trials (NCT04417621, NCT03905148, NCT04835805). Furthermore, pan-RAF inhibitors in combination with MEKi led to increased anti-tumor TILs and potentiated the effects of anti-PD-L1 checkpoint inhibition *in vivo*, providing rationale for testing this combination in patients (Hong, Piva, et al., 2021). Pre-clinical investigations are already anticipating mechanisms of pan-RAF inhibitor resistance, and ARAF activation has been uncovered as a potential resistance driver *in vitro* (Dorard et al., 2017; Yen et al., 2021). Overall, the MAPK pathway remains a primary therapeutic target in CM and using kinase inhibitors upstream or downstream of BRAF or MEK with existing targeted therapy is a rational combination approach.

### 4.2. Alternative survival pathways

Targeting survival pathways that act in parallel to MAPK signaling is another way to achieve potentially synergistic therapeutic effects with BRAFi and MEKi. Although less common than MAPK reactivation, constitutive activation of phosphoinositide 3-kinase/Akt serine threonine kinase/mammalian target of rapamycin (PI3K/AKT/mTOR) signaling occurs in a subset of BRAF and MEK inhibitor-resistant CMs, often through mutations that activate oncogenes (e.g., *AKT1*, *AKT3*, *PI3KCA*) or silence tumor suppressor genes (e.g., *PTEN*, *PHLPP1*) in this pathway (Shi, Hong, et al., 2014; Shi, Hugo, et al., 2014; Tran et al., 2021). Furthermore, *NRAS*-mutant CM has high basal activity of the PI3K/AKT/mTOR pathway due to constitutive RAS signaling (Petit et al., 2019; Posch et al., 2013). Genetic or pharmacologic silencing of AKT or PI3K, which is activated upstream of AKT, sensitizes CM cells to BRAF and MEK inhibition and delays acquired resistance in pre-clinical models (Atefi et al., 2015; Petit et al., 2019; Posch et al., 2013; Shi, Hong, et al., 2014; Tran et al., 2021). However, clinical trials combining a pan-AKT inhibitor or pan-PI3K inhibitor with BRAFi and/or MEKi showed limited responses and high toxicity in CM (Algazi et al., 2017; Algazi et al., 2018; Algazi et al., 2019). Patients with refractory melanoma brain metastasis may still stand to benefit from this combination approach given that brain metastases have increased expression of PI3K/AKT pathway genes relative to extracranial metastases (Chen et al., 2014; Fischer et al., 2019) and show high sensitivity to PI3K/AKT inhibition *in vivo* (Aasen et al., 2019; Amaral et al., 2020; Brastianos et al., 2015). A trial examining the efficacy of encorafenib and binimetinib plus a pan-PI3K inhibitor for refractory CM is ongoing (NCT02159066), and it remains to be seen if this combination is more effective than previous attempts at blocking PI3K/AKT.

Given the marginal clinical activity of PI3K/AKT inhibitors, more recent efforts have focused downstream of PI3K signaling on mTOR, which also drives targeted therapy resistance (Gopal et al., 2014; Wang, Zhang, et al., 2021). Despite being approved for various malignancies, early clinical trials with mTOR inhibitors (mTORi) in CM demonstrated limited efficacy when combined with BRAFi or MEKi (Subbiah et al., 2018; Tolcher et al., 2015), exhibiting toxicity and only some partial responses. mTOR is a component of both the mTORC1 and mTORC2 complexes, and most mTORi predominantly target mTORC1, leading to re-activation of PI3K/AKT signaling via uninhibited mTORC2 (Kim, Cook, & Chen, 2017). Combined therapy with a MEK inhibitor plus a dual PI3K/mTOR inhibitor is able to overcome this positive feedback, showing synergy *in vitro* and *in vivo* (Posch et al., 2013; Tran et al., 2021). However, this combination again had poor tolerability and only a 14% response rate in CM patients (Schram et al., 2018). Therapeutic strategies that prevent adaptive re-activation of PI3K/AKT/mTOR signaling continue to be developed. ATP-competitive mTORi that simultaneously inhibit both mTORC1 and mTORC2, and thereby avert rebound PI3K/AKT activation, are being tested in phase I and II clinical trials across cancer types, although none of these studies are being conducted in combination with MAPK-targeting agents (Tian, Li, & Zhang, 2019). Bi-steric mTORC1 inhibitors have been proposed as an alternative approach and a phase I clinical trial with one of these drugs is ongoing in patients with refractory solid tumors (NCT04774952). These inhibitors are highly selective for mTORC1 and completely abrogate 4EBP1 phosphorylation, thereby blocking protein translation and exerting potent anti-tumor effects without the induction of adaptive RTK signaling (Lee et al., 2021). Sensitivity to mTORi is also dependent on factors beyond the PI3K/AKT/mTOR pathway, such as the metabolic phenotype of CM cells (Gopal et al., 2014), and investigating additional drivers of response could provide clues as to which patients might benefit most from mTOR inhibitor therapy.

Targeted inhibition of survival pathways beyond PI3K/AKT/mTOR is also being tested in combination with BRAFi and/or MEKi in CM. Autophagy, a lysosomal degradation pathway that maintains nutrient homeostasis, promotes cell survival in MAPK targeted therapy-resistant cells and can be inhibited by hydroxychloroquine to overcome resistance (Amaravadi, Kimmelman, & Debnath, 2019; Ma et al., 2014). Preliminary clinical data from a phase I/II trial of hydroxychloroquine in combination with dabrafenib plus trametinib showed a good safety profile, a 48.2% 1-year PFS rate, and 11.2 months median PFS in *BRAF*-mutant CM patients (Mehnert et al., 2022). These findings have launched a multi-institutional randomized phase II trial comparing dabrafenib plus trametinib with either hydroxychloroquine or placebo in stage IIIC and stage IV *BRAF* V600 mutant CM (NCT04527549). Results from a similar phase I/II trial are pending (NCT03754179), and hydroxychloroquine in combination with trametinib is also being tested in *NRAS*-mutant CM (NCT03979651). In UM, inhibiting the MAPK pathway plus autophagy displayed synergy *in vitro* and *in vivo* (Truong et al., 2020), suggesting that this approach can be translated across melanoma subtypes, including to tumors that are relatively impervious to targeted therapy. Although blocking MAPK signaling has been a major focus of kinase inhibitors, targeting parallel cell survival pathways that get upregulated with BRAFi and MEKi resistance is a viable therapeutic strategy.

### 4.3. Cell cycle kinases

Dysregulation of cell cycle progression is a hallmark of cancer. Aberrant cell cycle control is common in CM and is driven by mutations in *BRAF* and *NRAS*, which occur in >75% of CMs and lead to cell cycle progression downstream of constitutive MAPK pathway activation, as well as by genetic alterations in *CDKN2A*, which encodes p16^INK4a^, a negative regulator of cell cycle progression, and increased expression of proteins such as Cyclin D1 and CDK4, which promote G1-S cell cycle progression (Sheppard & McArthur, 2013). Alterations in *CDKN2A* occur in ~60–70% of *BRAF*, *NRAS*, and *NF1*-mutant CMs, whereas wild-type CM has less frequent *CDKN2A* mutations (~40%) but copy number amplifications of *CDK4* (~15%) and *CCND1* (~10%), the gene that encodes Cyclin D1, are more common (Genomic Classification of Cutaneous Melanoma, 2015). Efforts to target the cell cycle are of particular interest in *NRAS*-mutant CM, where cell cycle dysregulation has been shown to be NRAS-dependent (Kwong et al., 2012), and in targeted therapy-resistant CM, which is associated with alterations in cell cycle proteins (Smalley et al., 2008; Yadav et al., 2014).

CDK4/6i (palbociclib, ribociclib, abemaciclib) act by preventing the CDK4/6-Cyclin D1 complex from phosphorylating retinoblastoma protein, leading to cell cycle arrest. Palbociclib has been approved for clinical use in ER-positive/HER2-negative breast cancer combined with an aromatase inhibitor, highlighting this therapy’s translational potential. In CM, the combination of MEKi plus CDK4/6i reduces tumor growth *in vitro* and *in vivo*, across genomic subtypes, and in BRAFi/MEKi-resistant patient-derived xenografts (Nassar et al., 2021; Posch et al., 2018; Teh et al., 2016). Several clinical trials have evaluated the safety and efficacy of combining CDK4/6i with BRAFi or MEKi in *BRAF*-mutant (NCT02202200, NCT01777776) or wild-type (NCT02065063, NCT01781572) CM. Response to triple therapy with ribociclib plus encorafenib and binimetinib was lower in patients who had previously progressed on targeted therapy (5.3% objective response rate) (Dummer et al., 2020) than in those who were treatment-naïve (52.4% objective response rate) (Ascierto et al., 2017). A trial examining palbociclib plus encorafenib and binimetinib in patients who are either therapy-naïve or have refractory disease is ongoing (NCT04720768). In addition to tumor-intrinsic responses, several groups have evaluated the effects of CDK4/6i on the TIME in CM. CDK4/6i demonstrate both anti- and pro-tumor immunity, leading to increased T-cell activation, infiltration, and memory (Jerby-Arnon et al., 2018; Lelliott et al., 2021; Teh et al., 2020), but also depleting myeloid cell populations necessary for T-cell priming and a pro-inflammatory response (Lelliott et al., 2021). CDK4/6i can also increase PD-L1 expression in tumor cells *in vivo* (Zhang et al., 2018). Furthermore, combining CDK4/6i with BRAFi and MEKi *in vivo* enhanced anti-tumor responses mediated by adoptive T-cell transfer (Lau et al., 2021). The immunogenicity of CDK4/6i opens up the possibility of combining these inhibitors with immunotherapy to potentiate anti-tumor activity.

Despite encouraging results with CDK4/6i, resistance to these therapies has been reported across cancer types (Teh & Aplin, 2019). In CM, CDK4/6 inhibitor monotherapy is limited by upregulation of the MDM2-p53 and PRMT5-MDM4-p53 signaling axes (AbuHammad et al., 2019; Vilgelm et al., 2019), while resistance to combined CDK4/6i plus MEKi in *NRAS*-mutant melanoma is driven by genetic alterations in RTKs, *RAF*, *RAS*, and the PI3K/AKT signaling pathway (Hayes et al., 2019; Romano et al., 2018; Teh et al., 2018). However, these resistance mechanisms may represent additional therapeutic targets. For example, MDM2 inhibitors (Vilgelm et al., 2019) or PRMT5 inhibitors (AbuHammad et al., 2019), could be used in combination with CDK4/6i to prolong response. In CDK4/6 inhibitor plus MEK inhibitor resistant tumors, targeting either mTOR or S6K1, which are activated downstream of PI3K, re-sensitized tumors to the combination therapy (Romano et al., 2018; Teh et al., 2018).

Additional therapeutic strategies to consider for cell cycle inhibition include CDK4/6-targeting proteolysis-targeting chimeras (PROTACs) and alternative cell cycle checkpoint kinase inhibitors. PROTACs are bi-functional small molecules that contain both a ligand-binding protein and a ubiquitin ligase. Once bound to a target of interest, they facilitate ubiquitination and subsequent degradation of the target (Verma, Mohl, & Deshaies, 2020). CDK4/6-targeting PROTACs show activity against many cancer types, including CM (Wu et al., 2021), and are effective even when target proteins are mutated or overexpressed (Su et al., 2019). PROTACs targeting the estrogen receptor and the androgen receptor are in clinical trials for breast and prostate cancer (NCT04072952, NCT03888612), respectively, highlighting their translational potential. Aurora kinase A/B, PLK1, WEE1, and CHK1 act as cell cycle checkpoints, pausing cell cycle progression in S or G2 phase when DNA damage is detected, allowing for DNA damage repair (Otto & Sicinski, 2017). Inhibition of cell cycle checkpoints is thought to induce cell death via an accumulation of DNA damage and replicative stress. The aforementioned checkpoint proteins have been identified as potential targets in both BRAFi-sensitive and BRAFi-resistant melanoma models (Bonet et al., 2012; Hwang, Adhikary, Eckert, & Lu, 2018; Margue et al., 2019; Pathria et al., 2016). An innovative approach using a dual-target inhibitor designed to block BRAF and Aurora kinase B activity simultaneously was shown to be effective in BRAFi-sensitive and -resistant CM (Chang et al., 2020). Furthermore, combining WEE1 inhibitors with CHK1 inhibitors induces more CM cell death than either drug alone (Magnussen et al., 2015; Margue et al., 2019). Although pilot clinical studies are required to validate the safety and efficacy of these drugs in patients, there is evolving rationale for combining BRAFi and MEKi with these novel cell cycle kinase inhibitors in CM.

## 5. Targeted kinase inhibitors and combinations in rare melanoma subtypes

The development of targeted therapies for AM, UM, CJM, and MM has been limited due to the low incidence of these melanomas and their distinct genetic profiles, histopathology, and disease progression. However, kinase inhibitors are a class of drugs that have been trialed and hold promise as a therapeutic strategy across rare melanoma subtypes (Fig. 2).

### 5.1. Uveal melanoma

Arising within the uveal tract of the eye, UM has a unique genetic profile. Current clinical guidelines for primary UM include complete removal of the eye (enucleation) or plaque brachytherapy (Barker & Salama, 2018). However, even after successful treatment, approximately half of all UM patients develop metastasis, predominantly in the liver (Bakalian et al., 2008), and experience median OS of approximately one year (Rantala, Hernberg, & Kivelä, 2019). Although the MAPK pathway is downstream of *GNAQ/11* driver mutations in UM, patients experience poor responses to targeted kinase inhibitors (Carvajal et al., 2014; Carvajal et al., 2018). Treatment of UM with ipilimumab plus nivolumab has overall response rates of 12–18% and leads to a modest improvement in median OS (up to 12–19 months) (Pelster et al., 2021; Piulats et al., 2021), while overall response rates to immune checkpoint inhibitor monotherapy range between 0 and 30% (Heppt et al., 2017). Thus, the same ICi used in CM have largely modest effects in UM.

Targeting other immune checkpoints in addition to PD-1 and CTLA-4 may be more effective in UM. Pre-clinical studies identified increased expression of LAG-3 on the TILs of metastatic and high-risk UM cases, presenting a new immunotherapeutic option for UM (Durante et al., 2020; Karlsson et al., 2020). Already, phase I clinical trials are examining the efficacy of relatlimab in combination with nivolumab (NCT04552223). In addition, XmAb^®^22841, a monoclonal bispecific antibody that targets both LAG-3 and CTLA-4, is being studied in a phase I clinical trial in combination with pembrolizumab (NCT03849469), an approach that effectively inhibits three of the most essential immune checkpoints in UM.

As an alternative to immune checkpoint inhibition, T-cell redirecting therapy, which brings cancer cells and T-cells in close proximity to one another and has been successfully implemented in hematological malignancies, is now being tested in UM patients. A phase III clinical trial demonstrated that Tebentafusp, a bispecific protein which utilizes melanocyte marker gp100 as a target, increases median OS up to 21 months, findings that led to FDA approval of this therapy (Nathan et al., 2021). However, this therapeutic option is limited to patients who express a specific human leukocyte antigen (HLA-A*02:01) that can be recognized by the therapy (Nathan et al., 2021). Mimicking the mechanism of Tebentafusp, another T-cell redirecting therapy using TYRP1 as a marker is in an early clinical trial (NCT04551352), and additional T-cell redirecting bispecific proteins that recognize other targets (e.g., PRAME) are being developed (Bunk et al., 2019). Given that immunotherapies have only modestly improved survival in UM, there is an urgent unmet need to provide novel targeted approaches for these patients.

Immunotherapy has not been as successful in UM as compared to CM, effects that correlate with a lack of ultraviolet signature mutations and low mutational burden compared to CM (Furney et al., 2013; J. Y. Kim et al., 2019). Additionally, BRAFi, which are frequently used for CM, are not effective in UM due to the presence of wild-type BRAF (Rimoldi et al., 2003). Instead, approximately 98% of all UM cases have mutually exclusive somatic activating mutations in one of four G-protein-coupled receptor (GPCR) components: *GNA11*, *GNAQ*, *PLCB4*, or *CYSLTR2* (Chua et al., 2017; Moore et al., 2016). These mutations initiate UM by constitutively activating the PLC/PKC/MAPK and Trio-Rho/Rac/YAP pathways (Feng et al., 2014; Li et al., 2019). Primary GPCR component mutations are often followed by loss of heterozygosity in chromosome 3 and missense mutations in BRCA1-associated protein 1 (*BAP1*) (Shain et al., 2019). Up to 90% of metastatic patients have *BAP1* mutations (Harbour et al., 2010; Karlsson et al., 2020). Thus, targeting these mutations directly, or inhibiting the downstream pathways and kinases they activate, could be an effective therapeutic strategy in UM. However, targeted monotherapy has not always proven successful in the clinic. For example, even though activating mutations in *GNAQ/GNA11*, which code for heterotrimeric G-proteins Gαq/11, lead to increased activity of the MAPK pathway in UM, MEK inhibition alone or in combination with dacarbazine, an alkylating chemotherapeutic agent, did not show any OS benefit (Carvajal et al., 2014; Carvajal et al., 2018). This limited response occurs because, while activating mutations in Gαq/11 are necessary for tumor formation, they are not related to the risk of metastasis or overall patient outcomes (Koopmans et al., 2013; Onken et al., 2008). Furthermore, the release of growth factors from the tumor microenvironment facilitates drug resistance and prevents complete inhibition of growth signaling upon targeted kinase monotherapy (Cheng et al., 2015; Cheng et al., 2017). Given that multiple effector pathways drive UM, and responses to MEK inhibitor monotherapy have been limited, there is a strong rationale for combining targeted kinase inhibitors in metastatic UM.

#### 5.1.1. MAPK and PKC pathway kinases

One approach to improving the anti-tumor effects of targeted kinase therapy is to enhance inhibition of the MAPK pathway, either by suppressing extrinsic drug resistance mechanisms that arise from the tumor microenvironment or by blocking kinases that activate MAPK signaling. Metastatic UM occurs predominantly in the liver, and due to an abundance of hepatocyte growth factor (HGF), these tumors have increased activation of the RTK, cMET (Cheng et al., 2017). Pre-clinical studies identified HGF as a driver of resistance to MEK inhibitor monotherapy, which can be overcome by directly targeting the cMET receptor (Cheng et al., 2017). HGF mediates resistance via sustained upregulation of the PI3K/mTOR pathway, which is downstream of cMET (Cheng et al., 2017). MEKi in combination with mTORi have shown synergistic effects in pre-clinical models of UM (Amirouchene-Angelozzi et al., 2014), and combination therapy targeting both the PI3K/mTOR and PKC/MAPK axes with a PI3K and pan-PKC inhibitor was effective in reducing xenograft tumor growth (Musi, Ambrosini, de Stanchina, & Schwartz, 2014). However, the use of these inhibitors was not translatable to a clinical setting due to either intolerability in other solid tumors (Tolcher et al., 2015) or limited clinical activity in UM (Shoushtari et al., 2021). Future clinical trials may want to consider MEKi plus direct inhibition of cMET. An additional approach explored the effects of targeting the receptor of another liver secreted growth factor, insulin-like growth factor 1 (IGF1), which activates AKT (Cheng et al., 2015). Although a phase II clinical trial showed that an IGF1 receptor inhibitor did not reduce UM tumor progression, it was well tolerated (Mattei et al., 2020) and could thus be used in combination with a MEK inhibitor. The liver tropism of metastatic UM provides an opportunity to strategically inhibit liver growth factor-dependent receptors in combination with the MAPK pathway. Furthermore, due to the incomplete inhibition of ERK1/2 signaling with MEKi alone, there is a strong rationale to combine targeted therapies that affect the same pathway. UM is highly dependent on MAPK activity driven by PKC signaling (Ma, Weng, Bastian, & Chen, 2021), and combining MEKi and PKC inhibitors is synergistic (Sagoo, Harbour, Stebbing, & Bowcock, 2014). In phase I clinical trials, PKC inhibitors showed a favorable safety profile in UM (Kapiteijn et al., 2019; Piperno-Neumann et al., 2020) and clinical trials testing PKC inhibitors as neoadjuvant and adjuvant therapy (NCT05187884), or in combination with a MEK or cMET inhibitor (NCT03947385), have been initiated. Combining MAPK targeted therapies with inhibitors against parallel signaling pathways is a strategy that may reduce UM tumor growth.

#### 5.1.2. Trio-Rho/Rac/YAP pathway kinases

Pathways downstream of oncogenic G-protein signaling include the PLCβ/PKC/MAPK pathway and the Trio-Rho/Rac/YAP pathway. Just as *GNAQ/GNA11* mutations are not indicative of overall patient survival outcomes, YAP activity may not be essential for UM survival (Kim et al., 2020). However, a synthetic lethal gene interaction network elucidated that downstream of Trio-Rho/Rac, focal adhesion kinase signaling was important for YAP activity and could be therapeutically targeted (Feng et al., 2019). This provides an additional pathway downstream of Gαq that can be targeted in parallel to the MAPK pathway. In fact, combining focal adhesion kinase inhibitors (FAKi) with MEKi resulted in cytotoxic effects, and more drastic tumor regression was seen *in vivo* with the combination, as compared to treatment with either inhibitor alone (Paradis et al., 2021). The safety of FAKi has already been tested in other cancer types (Mohanty et al., 2020), and they are now in clinical trials for UM as monotherapy and in combination with cobimetinib (NCT04109456) or with VS-6766 (NCT04720417, NCT03875820), a dual RAF/MEK inhibitor that received FDA breakthrough therapy designation for low-grade serous ovarian cancer (Ishii et al., 2013). Elucidating the pathways downstream of mutations driving UM tumorigenesis has led to the identification of druggable targets that can be inhibited alongside the MAPK pathway. Additional pre-clinical studies would be helpful in developing other synergistic combination therapies in UM.

#### 5.1.3. Directly targeting Gα-q/Gα−11 signaling

FR900359 and YM-254890 are two naturally derived depsipeptides that target Gαq/11 and have therapeutic potential in treating UM (Lapadula et al., 2019; Ma et al., 2021; Onken et al., 2018). *In vitro*, these inhibitors block Gαq/11 signaling and downstream cell cycle progression at low concentrations and induce cell death at high concentrations in mutant *GNAQ/GNA11* UM cell lines. They also molecularly reprogram cells to undergo melanocyte re-differentiation (Annala et al., 2019; Onken et al., 2021). Both wild-type and mutant forms of Gαq/11 are targeted by FR900359 and YM-254890, which limits the therapeutic window of these inhibitors when given systemically. *In vitro*, these inhibitors lead to incomplete inhibition of the MAPK pathway, potentially due to the activation of parallel survival pathways, such as via ERBB3/HER3 upregulation (Lapadula et al., 2019). *In vivo*, a therapeutically effective dose of FR900359 reduced ERK1/2 phosphorylation levels by only 30% (Onken et al., 2021). Similarly, inhibition with YM-254890 alone did not abolish ERK1/2 phosphorylation, and rebound ERK1/2 activation was observed in some cell lines after 24 h of treatment (Hitchman et al., 2021). Instead, the combination of YM-254890 with trametinib proved to have a synergistic effect on inhibiting tumor growth (Hitchman et al., 2021). Thus, due to incomplete inhibition of ERK1/2 phosphorylation and possible off-target effects, a combination therapy approach is warranted to achieve maximal therapeutic benefit and mitigate side effects with Gαq/11 inhibitors. There is also the potential to leverage the molecular reprogramming induced by these therapies. For example, in one UM cell line, an increase in gp100 expression was observed after FR900359 inhibition (Annala et al., 2019). Thus, Tebentafusp, which binds gp100 and redirects T-cells to a tumor site, could have increased efficacy when combined with Gαq/11 inhibitors. Further experiments are necessary to determine if FR900359 increases gp100 antigen presentation *in vivo* and if pretreatment with FR900359 can enhance the effects of Tebentafusp. Direct Gαq/11 targeting in combination with kinase inhibitors holds a lot of potential in treating UM, especially if systemic side effects can be mitigated by targeted drug delivery and rational drug combinations.

#### 5.1.4. Epigenetic inhibitors

The use of transcription-modifying agents is especially pertinent to UM because inactivating *BAP1* mutations occur in 35% of all UM patients (Moore et al., 2016), and *BAP1* loss is found in the majority of patients with metastasis or at elevated risk for metastasis (Karlsson et al., 2020). BAP1 is a deubiquitylating (DUB) enzyme known for its role in DUB activity of histone H2A (Masclef et al., 2021). It also plays an indirect role in H3K27 acetylation by regulating HDAC4 levels (Kuznetsov et al., 2019). Loss of BAP1 increases histone deacetylase levels, and a pre-clinical drug screen identified that pan-histone deacetylase inhibitors (HDACi), such as quisinostat, are effective at inhibiting UM growth (Kuznetsoff et al., 2021). In UM, HDACi were able to reverse the adaptive survival mechanisms of increased YAP and AKT signaling after MEK inhibition, and combining a pan-HDAC inhibitor, panobinostat, with trametinib decreased tumor volume in animal models more than either drug alone (Faião-Flores et al., 2019). A phase II clinical trial examining a MEK inhibitor in combination with an HDAC inhibitor is ongoing (NCT05170334). Similarly, decitabine, a DNA methyltransferase inhibitor, in combination with a MEK inhibitor increased proapoptotic protein expression and enhanced growth inhibition more than either drug alone (Gonçalves et al., 2020). Kinases have also been targeted to overcome resistance to epigenetic inhibitors. Bromodomain and extra-terminal motif protein inhibitors (BETi) were used in a clinical trial against various solid tumors, including hepatic UM metastases, but showed minimal efficacy, and patients quickly faced disease progression (Chua et al., 2019; Patnaik et al., 2018). Tumor biopsies revealed that increased expression of FGFR, an RTK, mediated BETi resistance in metastatic UM (Chua et al., 2019). *In vivo* experiments suggest that FGFR could be directly targeted in combination with BETi to reduce tumor growth (Chua et al., 2019). Future clinical trials could consider testing FGFR inhibitors plus BETi for UM. Overall, epigenetic modifications play an important role in UM, and inhibiting these processes along with MAPK signaling is a promising therapeutic strategy.

#### 5.1.5. Cell cycle kinases and metabolism

Cancers have distinct metabolic profiles from normal tissue and metabolic adaptations can drive resistance to targeted therapy (Avagliano et al., 2020). In the context of UM, pre-clinical studies show that CDK4/6i and MEKi effectively reduce cell growth *in vitro* (Teh et al., 2020). However, in an *in vivo* model, these inhibitors were ineffective. Oxidative phosphorylation was increased after targeted inhibition of the cell cycle, resulting in increased susceptibility to oxidative phosphorylation inhibitors (Teh, Purwin, et al., 2020). Metabolic adaptations also commonly occur in UM as a result of *BAP1* mutations and loss of chromosome 3 (Chattopadhyay et al., 2019; Han, Purwin, & Aplin, 2021). For example, increased dependency on the glycolytic pathway and altered energy-sensing abilities are potential vulnerabilities that can be targeted to reduce UM cell growth (Chua et al., 2022; Han et al., 2022). Furthermore, *BAP1* mutant UM cases have distinct metabolic subtypes, classified according to their oxidative phosphorylation activity, and respond differentially to metabolic inhibitors (Han et al., 2021). Identification of these subtypes provides further rationale to combine metabolic inhibitors with kinase inhibitors in UM.

### 5.2. Conjunctival melanoma

The rarity of CJM has meant that no standard of care has been developed for these melanomas. Surgical excision of primary tumors is conducted, followed by adjuvant chemotherapy or radiotherapy if indicated, and monitoring for disease recurrence or metastasis (Grimes, Shah, Samie, Carvajal, & Marr, 2020; Jain et al., 2021). After primary treatment, 66% of cases eventually develop local recurrence, and the 10-year OS rate is 62% (Brouwer et al., 2021). Although CJM arises from the conjunctival epithelium of the eye, it has a mutational profile most similar to CM and, of the rare melanomas, has shown the most promising response to targeted BRAFi and MEKi *in vitro* and in individual patient case studies (Brouwer et al., 2021; Cao et al., 2017; Griewank et al., 2013). CJM cell lines have also shown sensitivity to PI3K/AKT pathway inhibitors (El Zaoui et al., 2019), and these drugs synergized with MEKi *in vitro* (Cao et al., 2017). Additionally, ICi produced promising responses within small cohorts of patients (Hong et al., 2021; Sagiv et al., 2018). However, none of these findings have been validated by a clinical trial. Further analysis of what drives CJM recurrence and metastasis is necessary to identify treatments that can produce sustainable responses in patients.

### 5.3. Acral lentiginous melanoma and mucosal melanoma

AM accounts for 2–3% of all melanomas, but in some countries in Latin America, Africa, and Asia, AM makes up the majority of melanoma cases (Basurto-Lozada et al., 2021). MM makes up approximately 1% of all melanoma cases, and its incidence has remained fairly stable (Chang, Karnell, & Menck, 1998; Tyrrell & Payne, 2018). Clinical approaches for AM and MM include surgical excision of the primary tumor, while treatment options for metastatic disease are lacking (Swetter et al., 2019; Tyrrell & Payne, 2018), and survival rates are lower than for patients with CM. Patients are rarely candidates for BRAFi and MEKi, given that few of these tumors exhibit *BRAF* mutations (Hayward et al., 2017; Newell et al., 2020; Tyrrell & Payne, 2018), and *BRAF* V600 mutant AM responds poorly (Jamerson, Rebecca, & Aguh, 2021). Combined immune checkpoint inhibition in AM and MM has reported some response, including an objective response rate of ~40% and approximately 6 months median PFS in MM (D’Angelo et al., 2017); however, this remains lower than that seen in CM (Cho et al., 2021; Li, Kan, Zhao, Sun, & Bai, 2020). Alternative approaches using targeted kinase inhibitors are being studied in AM and MM, and hold promise for these rare melanoma subtypes.

In contrast to non-acral cutaneous melanoma, AM and MM tumors show frequent activation of *KIT* (Curtin, Busam, Pinkel, & Bastian, 2006; Hayward et al., 2017), and monotherapy targeting the c-KIT RTK is a potential treatment. Early trials with imatinib showed high response rates in *KIT*-mutant, but not *KIT*-amplified, melanomas (Carvajal et al., 2011; Guo et al., 2011; Hodi et al., 2013). Nilotinib was also deemed safe and effective for *KIT*-mutant rare melanomas (Carvajal et al., 2015; Guo et al., 2017). A recent pooled analysis found a 14% and 22% objective response rate for c-KIT inhibitors trialed in MM and AM, respectively, and the highest response occurred with nilotinib (Steeb et al., 2021). Response to c-KIT inhibition is mutation-dependent (Sabbah et al., 2021), and drug resistance arises via various mechanisms, including activation of MAPK and PI3K/AKT signaling or cMET (Carlino, Todd, & Rizos, 2014; Oba et al., 2018). Combining c-KIT inhibitors with targeted therapies that block these resistance mechanisms could be effective. Trials with PLX3397, a novel c-KIT inhibitor (NCT02071940), and anlotinib, a pan-RTK inhibitor that also targets c-KIT (NCT03991975, NCT05087602), are ongoing. In addition to frequent c-KIT activation, genomic aberrations in CDK4 pathway related genes, such as *CDK4*, *CCND1*, and *CDKN2A*, have been identified in more than 80% of AM patients (Kong et al., 2017), and CDK4/6i monotherapy had a favorable safety profile in a small phase II clinical trial (Mao et al., 2021). Aberrant CDK4 pathway activity has also been linked to resistance to ICi in AM (Yu et al., 2019), and given that CDK4/6i modulate the TIME (Lelliott, McArthur, Oliaro, & Sheppard, 2021; Zhang et al., 2018), there is strong rationale for combining CDK4/6i with immunotherapy in AM. As with UM and CJM, the discovery of additional druggable targets is warranted so that durable response and survival rates can be achieved in patients.

## 6. Concluding remarks and future perspectives

Targeted kinase inhibitors have played an important role in the history of melanoma and will continue to do so in the future. BRAFi and MEKi have improved patient survival in *BRAF*-mutant CM, showing faster and higher, albeit less durable, response rates than ICi. In some cases, they are better tolerated than ICi and can particularly benefit patients who are symptomatic or have a high tumor burden. This review highlights how the efficacy of targeted kinase inhibitors continues to be enhanced via their repurposing for novel therapeutic applications, the identification of rational combinations with existing drugs, and the discovery of novel targets in melanoma. Already, MAPK targeting agents are approved in the adjuvant setting, and clinical trials in a neoadjuvant setting are underway. The use of immunotherapies is becoming more widespread, and BRAFi and MEKi are at the forefront of this field as some of the first small molecule inhibitors being tested in various sequencing combinations with ICi. This sets a precedent for studying the effects of new therapies on the TIME, so that they can be leveraged in order to enhance anti-tumor immune responses. Finally, new targeted kinase inhibitors and combination strategies are active in clinical trials in both CM and rare melanoma subtypes. This is particularly important for CM patients with intrinsic or acquired resistance to BRAFi/MEKi, or those who fail to respond to ICi, as they have limited treatment options. Patients with rare melanoma subtypes have access to even fewer therapeutic strategies, but promisingly, targeting kinases that drive tumorigenesis and metastasis, such as FAK, PKC, or cell cycle regulators, has shown therapeutic potential.

The future of kinase inhibitors partly depends on the identification of novel targets. Current targeted kinase inhibitor therapies focus mostly on known tyrosine kinases, yet there are upwards of 600 total kinases in the human genome (Essegian, Khurana, Stathias, & Schürer, 2020). New large-scale screening methods can unveil kinases within this ‘dark kinome’ that are involved in tumor initiation and drug resistance or that can serve as prognostic markers (Essegian et al., 2020). Large-scale quantitative proteomic screens identified understudied kinases as therapeutic targets in high-grade serous ovarian carcinoma and triple-negative breast cancer (Duncan et al., 2012; Kurimchak et al., 2020), and could be used to identify functionally relevant kinome reprogramming that occurs in melanoma. A drawback of this approach and other bulk screens is that it fails to capture intra-tumoral heterogeneity. Single-cell RNA-sequencing (scRNA-seq) could help identify transcript levels of kinases that drive clonal evolution or transcriptional reprogramming between phenotypic cell states, including those that lead to therapy resistance or metastasis. CM has recently gathered large scRNA-seq datasets from both malignant and non-malignant cells that are either treatment-naïve or treated with targeted therapy or immunotherapy (Fattore, Ruggiero, Liguoro, Mancini, & Ciliberto, 2019; Jerby-Arnon et al., 2018; Rambow et al., 2018). In UM, scRNA-seq of malignant and non-malignant cells has been conducted exclusively in the context of treatment-naïve tumors and in 3 metastatic samples (Durante et al., 2020; Karlsson et al., 2020; Pandiani et al., 2021). In other rare melanoma subtypes, only whole-genome sequencing data has been collected (Newell et al., 2019; Newell et al., 2020). Thus, data from ‘dark kinome’ screens and scRNA-seq holds promise for the development of novel targeted kinase inhibitor therapies.

In addition to identifying new targets, existing targeted kinase inhibitors can be redeveloped as dual-acting drugs with increased specificity and enhanced delivery mechanisms. Dual-acting inhibitors could reduce the side effects associated with combination therapies while still targeting multiple nodes within a signaling pathway. For example, a new MEK inhibitor currently in clinical trials for metastatic UM (NCT04720417) simultaneously inhibits RAF feedback activity and could be tested in other melanoma subtypes. Furthermore, some RTK inhibitors target pan-RTKs and could be used against heterogeneous populations of cells within a single tumor. Genetic testing can aid in the identification of which pan-RTK inhibitor would be most specific to a tumor, and this type of personalized medicine approach is already being tested in CM and other solid tumors (NCT02465060, NCT02159066). Targeted kinase inhibitors with toxic safety profiles could also be coupled with novel molecular or clinical drug delivery systems to reduce off-target effects. Nanoparticle delivery mechanisms, including ADCs, are under investigation across cancer types and could theoretically contain a combination of inhibitors (Yu et al., 2022). ADCs leverage increased expression of certain RTKs or molecular markers to deliver site-directed therapy. Already, ADCs are approved for breast cancer and are in clinical trials for CM (NCT02988817, NCT05135715). Novel c-KIT targeting ADCs trialed in other solid tumors could be used for metastatic UM, AM, MM, and a subset of CM that have high expression of c-KIT (Kim et al., 2022). Another clinical example of site-directed therapy is percutaneous hepatic perfusion, a minimally invasive drug delivery system that can reduce systemic side effects and is in clinical trials for metastatic UM to the liver (Karydis et al., 2018) (NCT02678572). Site-directed therapy with targeted kinase inhibitors is thus a potential means of achieving anti-tumor effects without compromising patient safety. In summary, targeted kinase inhibitors have revolutionized melanoma treatment. There is great potential for kinase inhibitors to continue to benefit patients in the future through the steady discovery of novel targets, applications, and drug delivery mechanisms.

## Figures and Tables

**Table 1:** Selected Clinical Trials using BRAFi and/or MEKi in adjuvant and neoadjuvant applications for CM.

Studies with Reported Outcomes							
Trial, NCT Number	Patient Population		Treatment Schedule	Study Design		Outcomes	Reference
COMBI-AD, NCT01682083	Resected stage III, BRAF V600 mutant CM		12-months adjuvant dabrafenib + trametinib vs. double placebo	Randomized, double-blind, placebo-controlled phase III		Median relapse-free survival at 60 months median follow-up: not reached vs. 16.6 months 5-year relapse-free survival rate: 52% vs. 36%; 5-year distant metastasis-free survival rate: 65% vs. 54%	(Dummer, Hauschild, et al., 2020)
Combi-Neo, NCT02231775	Resectable stage III or oligometastatic stage IV, BRAF V600 mutant CM		8-weeks neoadjuvant dabrafenib + trametinib, followed by surgery and up to 44 weeks of adjuvant dabrafenib + trametinib vs. standard of care adjuvant therapy	Randomized, open-label, phase II		Median event-free survival at 18.6 months median follow-up: 19.7 months vs. 2.9 months; trial stopped early due to significantly improved outcomes in the neoadjuvant arm	(Amaria, Prieto, et al., 2018)
NeoCombi, NCT01972347	Resectable stage IIIB-C, BRAF V600 mutant CM		12-weeks neoadjuvant dabrafenib + trametinib, followed by surgery and 40 weeks of adjuvant dabrafenib + trametinib	Single-arm, open-label, phase II		Median relapse-free survival at 27 months median follow-up: 30.6 months with complete pathological response vs. 18.0 months non-complete pathological response	(Long et al.,2019)
Ongoing Studies							
Trial, NCT Number		Patient Population	Treatment Schedule		Study Design	Status	
NCT02036086		Unresectable, stage IIIB-C, BRAF V600 mutant CM	8-weeks neoadjuvant vemurafenib + cobimetinib, followed by surgery and adjuvant vemurafenib + cobimetinib		Single-arm, open-label, pilot phase II	Active, not recruiting	
NCT04741997		Stage IIIB-D or stage IV, BRAF V600 mutant CM	24-weeks neoadjuvant encorafenib + binimetinib, followed by surgery and adjuvant encorafenib + binimetinib vs. 24-weeks neoadjuvant encorafenib + binimetinib, followed by surgery and adjuvant nivolumab		Randomized, open-label, pilot phase I	Recruiting	
NCT04221438		Resectable, stage IIIB-D, BRAF V600 mutant CM	8-weeks neoadjuvant encorafenib + binimetinib followed by surgery and adjuvant encorafenib + binimetinib		Single-arm, open-label, phase II	Not yet recruiting	
PREMIUM, NCT05097378		Resectable stage III or oligometastatic stage IV, BRAF V600 mutant CM	8-weeks neoadjuvant encorafenib + binimetinib followed by surgery and up to 44-weeks adjuvant encorafenib + binimetinib vs. surgery and standard of care adjuvant therapy		Randomized, open-label, phase II	Not yet recruiting	
Neo Trio, NCT02858921		Resectable stage IIIB-C, BRAF V600 mutant CM	6-weeks neoadjuvant: dabrafenib + trametinib + sequential pembrolizumab vs. dabrafenib + trametinib + concurrent pembrolizumab vs. pembrolizumab alone; all arms followed by surgery and adjuvant pembrolizumab		Randomized, open-label, phase II	Active, not recruiting	
NeoACTIVATE, NCT03554083		Resectable stage III CM	12-weeks neoadjuvant atezolizumab + tiroligumab, or atezolizumab + cobimetinib, or atezolizumab + vemurafenib + cobimetinib; all arms followed by surgery and adjuvant atezolizumab		Open-label, pilot phase I	Recruiting	
NEO-TIM, NCT04722575		Resectable stage III or oligometastatic stage IV CM	BRAF V600 mutant: 6-weeks neoadjuvant vemurafenib + cobimetinib vs. vemurafenib + cobimetinib + atezolizumab; BRAF wild-type: 6-weeks neoadjuvant cobimetinib + atezolizumab; all arms followed by surgery and adjuvant atezolizumab		Randomized, open-label, phase II trial	Recruiting	
ALTER-PATH NeoDT, NCT04310397		Resectable stage IIIB-D, BRAF V600 mutant CM	8-weeks neoadjuvant dabrafenib + trametinib, followed by surgery and adjuvant dabrafenib + trametinib or spartalizumab		Single-arm, open-label, pilot phase II	Active, not recruiting	

**Table 2:** Selected Clinical Trials using BRAFi/MEKi in combination with ICi for CM.

Concurrent with ICi					
Studies with Reported Outcomes					
Trial, NCT Number	Patient Population	Treatment Schedule	Study Design	Outcomes	Reference
KEYNOTE-022, NCT02130466	Unresectable stage III or stage IV, BRAF V600 mutant CM	Dabrafenib + trametinib + pembrolizumab vs. dabrafenib + trametinib + placebo	Randomized, double-blind, placebo-controlled phase II	Median PFS survival at 36.6 months median follow-up: 16.9 months vs. 10.7 months	(Ferrucci et al., 2020)
COMBI-i, NCT02967692	Unresectable stage III or stage IV, BRAF V600 mutant CM	Dabrafenib + trametinib + spartalizumab vs. dabrafenib + trametinib + placebo	Randomized, double-blind, placebo-controlled phase III	Median PFS at 27.2 months: 16.2 months vs. 12.0 months; study did not meet its primary endpoint	(Dummer et al., 2022)
NCT02027961	Unresectable stage III or stage IV CM	Cohort A: BRAF V600 mutant CM treated with dabrafenib + trametinib + durvalumab; Cohort B: BRAF wild-type CM treated with trametinib + concurrent durvalumab; Cohort C: BRAF wild-type CM treated with trametinib + sequential durvalumab	Open-label, dose escalation and expansion phase I	Median PFS for Cohort A at 20.8 months median follow-up: 11.2 months; for Cohort B at 22.1 months median follow-up: 4.9 months; Cohort C at 20.8 months median follow-up: 5.9 months	(Ribas et al., 2020)
IMspire150, NCT02908672	Unresectable stage IIIC or stage IV, BRAF V600 mutant CM	Vemurafenib + cobimetinib + atezolizumab vs. vemurafenib + cobimetinib + placebo	Randomized, double-blind, placebo-controlled phase III	Median PFS at 18.9 months median follow-up: 15.1 months vs. 10.6 months	(Gutzmer et al., 2020)
Ongoing Studies					
Trial, NCT Number	Patient Population	Treatment Schedule	Study Design	Status	
STARBOARD, NCT04657991	Unresectable stage IIIB-D or stage IV, BRAF V600 mutant CM	Encorafenib + binimetinib + pembrolizumab vs. placebo + pembrolizumab	Randomized, double-blind, placebo-controlled phase III	Recruiting	
TRIDENT/TRIBECA, NCT02910700	Unresectable stage III or stage IV, BRAF V600 mutant CM	Dabrafenib + trametinib + nivolumab, or trametinib + nivolumab, or encorafenib + binimetinib + nivolumab	Non-randomized, open-label, phase II	Recruiting	
Sequential with ICi					
Studies with Reported Outcomes					
Trial, NCT Number	Patient Population	Treatment Schedule	Study Design	Outcomes	Reference
DREAMSeq, NCT02224781	Unresectable stage III or stage IV, BRAF V600 mutant CM	Ipilimumab + nivolumab followed by crossover to dabrafenib + trametinib vs. dabrafenib + trametinib followed by crossover to ipilimumab + nivolumab	Randomized, open-label, phase III	2-year OS rate: 72% vs. 52%	(Atkinson et al., 2021)
SECOMBIT, NCT02631447	Unresectable stage III or stage IV, BRAF V600 mutant CM; BRAF V600 mutant MM	Ipilimumab + nivolumab followed by crossover to encorafenib + binimetinib vs. encorafenib + binimetinib followed by crossover to ipilimumab + nivolumab vs. 8-weeks induction with encorafenib + binimetinib, then ipilimumab + nivolumab followed by crossover to encorafenib + binimetinib	Randomized, open-label, phase II	2-year OS rate: 73% vs. 65% vs. 69% 2-year PFS rate: 65% vs. 46% vs. 57%	(Ascierto, Mandala, et al., 2021)
Induction before ICi					
Ongoing Studies					
Trial, NCT Number	Patient Population	Treatment Schedule	Study Design	Status	
ImmunoCobiVem, NCT02902029	Unresectable stage IIIB-C or stage IV, BRAF V600 mutant CM	3 months induction with vemurafenib + cobimetinib followed by vemurafenib + cobimetinib and crossover to atezolizumab vs. 3 months induction with vemurafenib + cobimetinib followed by atezolizumab and crossover to vemurafenib + cobimetinib	Randomized, open-label, phase II	Active, not recruiting	
EBIN, NCT03235245	Unresectable stage III or stage IV, BRAF V600 mutant CM; BRAF V600 mutant MM	12-weeks induction with encorafenib + binimetinib followed by ipilimumab + nivolumab vs. upfront ipilimumab + nivolumab	Randomized, open-label, phase II	Recruiting	
COWBOY, NCT02968303	Unresectable stage III or stage IV, BRAF V600 mutant CM	6-weeks induction with vemurafenib + cobimetinib followed by ipilimumab + nivolumab vs. upfront ipilimumab + nivolumab	Randomized, open-label, phase II	Recruiting	

## Abbreviations:

ACT: adoptive cell therapy
ADCs: antibody-drug conjugates
AJCC: American Joint Committee on Cancer
AKT: Akt serine threonine kinase
AM: acral lentiginous cutaneous melanoma
BAP1: BRCA1 associated protein 1
BETi: Bromodomain and extra-terminal motif protein inhibitors
BRAFi: BRAF inhibitors
CDK4/6i: cyclin-dependent kinase-4/6 inhibitors
CJM: conjunctival melanoma
CM: cutaneous melanoma of the non-acral skin
CTLA-4: cytotoxic T-lymphocyte-associated protein 4
DUB: deubiquitylating
ERK1/2i: ERK1/2 inhibitors
FAKi: focal adhesion kinase inhibitors
FDA: U.S. Food and Drug Administration
GPCR: G-protein-coupled receptor
HD-IL-2: high-dose interleukin-2
HDACi: histone deacetylase inhibitors
HGF: hepatocyte growth factor
ICi: immune checkpoint inhibitors
IFN: interferon-alpha-2b
IGF1: insulin-like growth factor 1
LAG-3: lymphocyte activation gene 3
MAPK: mitogen-activated protein kinase
MEKi: MEK inhibitors
MHC: major histocompatibility complex
MM: mucosal melanoma
mTOR: mammalian target of rapamycin
mTORi: mTOR inhibitors
OS: overall survival
PD-1: programmed death protein 1
PD-L1: programmed death ligand-1
PFS: progression-free survival
PI3K: phosphoinositide 3-kinase
PROTACs: proteolysis-targeting chimeras
RTKs: receptor tyrosine kinases
scRNA-seq: single-cell RNA-sequencing
TILs: tumor-infiltrating lymphocytes
TIME: tumor immune microenvironment
T-VEC: talimogene laherparepvec
UM: uveal melanoma..
